# Development of Organocatalytic Darzens Reactions Exploiting the Cyclopropenimine Superbase

**DOI:** 10.3390/molecules29184350

**Published:** 2024-09-13

**Authors:** Carmine Lops, Lucia Pasquato, Paolo Pengo

**Affiliations:** Department of Chemical and Pharmaceutical Sciences, University of Trieste, Via Licio Giorgieri 1, 34127 Trieste, Italy; carmine.lops@hotmail.com

**Keywords:** Alfa-halo esters, α-halo carbonyl compounds, *α*,*β*-epoxy esters, carbon nucleophiles, organocatalysis

## Abstract

A truly organocatalytic approach to the Darzens reaction affording *α*,*β*-epoxy carbonyl compounds in good yields was developed taking advantage of the high basic strength and low nucleophilicity of cyclopropenimine superbases. The catalytic active free base can easily be generated in situ from its hydrochloride salt and maintained in the active deprotonated form by performing the reactions in a heterogeneous reaction system in the presence of excess potassium carbonate as a sacrificial base.

## 1. Introduction

The Darzens reaction, i.e., the condensation of aldehydes or ketones with α-halo carbonyl compounds in the presence of strong inorganic and/or organic bases is a non-oxidative approach to the preparation of *α*,*β*-epoxy esters and other *α*,*β*-epoxy carbonyl compounds. At variance with oxidation reactions, either metal catalysed [1,2,3,4,5,6,7,8] or organocatalytic [9,10,11,12,13,14,15], that require the forerunning preparation of *α*,*β*-unsaturated compounds, the Darzens reaction enables the formation of a new carbon-carbon bond and closure of the epoxide ring in a single synthetic step. This approach is remarkably advantageous since it may help shorten long synthetic routes and requires low-cost fragments. In view of that, and given the relevance of epoxides and *α*,*β*-epoxy carbonyl compounds as synthetic intermediates, refs. [16,17,18,19,20,21]. This reaction recently enjoyed a renewed interest, and some effort has been devoted to improving its synthetic applicability. This resulted in the development of a variety of different reaction conditions among which Phase Transfer Catalysis (PTC), ref. [22] and Lewis acid catalysis [23] are particularly relevant. However, in its base promoted versions—including those performed under PTC—the Darzens reaction leading to *α*,*β*-epoxy esters still represents a challenge. The major limitation is represented by the easy hydrolysis of the epoxyester formed in the very same conditions employed for the condensation [24]. Hydrolysis is also selective towards the *trans* isomer of the epoxyester and is significant even in the case of *t*-butyl esters [25]. This essentially limits the scope of Darzens reactions to pronucleophiles such as α-haloketones [26,27,28,29,30], α-chloroamides, refs. [31,32] or nitriles [32,33] as well as α-halosulfones [34,35,36].

While the most recent approaches to Darzens and Darzens-like reactions mainly rely on Lewis acid, and PTC catalysis, novel strategies based on exploiting supramolecular catalysis are emerging. As far as Lewis acid catalysis is concerned, Xie, Guo and co-workers recently developed an asymmetric Darzens reaction of isatins that provides access to spiro-epoxyoxindoles using Ni(acac)_2_ as the Lewis acid and imidazolidine-pyrroloimidazolone pyridine as ligand [23]. Highly enantioselective Darzens-like epoxidation of diazoesters with glyoxal derivatives could be achieved using a chiral boron–Lewis acid catalyst, allowing the asymmetric synthesis of trisubstituted α,β-epoxy esters. Ref. [37] In The field of PCT catalysis for Darzens reactions, the use of chiral phosphonium ions instead of quaternary ammonium ions has also been pursued in recent years. Indeed, Wang ad co-workers developed a highly efficient aza-Darzens cyclization between cyclic imines and α-halogenated ketones by employing a dipeptide-based chiral bifunctional phosphonium salt [38,39]. Catalytic approaches to Darzens reactions by supramolecular hosts used as nanoreactors have recently allowed three-component aza-Darzens reactions leading to aziridines to be carried out in water. The three components are aldehydes, anilines and substituted diazo esters; suitable supramolecular hosts were γ-cyclodextrins or metallacages [40,41,42,43]. Notwithstanding these achievements, the Darzens condensation involving α-halo esters still remains challenging.

We recently developed a base-promoted Darzens reaction in aprotic solvents—under non solvolytic conditions—involving the use of stoichiometric amounts of the charge-neutral Schwesinger bases P1-*t*-Bu and P4-*t*-Bu [44]. Although phosphazenes potentially have great utility, both the problems of their stability and difficulties of their preparation make the identification of alternative superbases for Darzens reactions an important goal. Most importantly an organocatalytic approach to this reaction, of which there is no example at present, would be highly desirable. Herein we describe the use of 2,3-bis(dicyclohexylamino)cyclopropenimines to fill this gap, taking advantage of its high basic strength that has been exploited in the development of enantioselective Mannich [45], Michael [46,47,48], and [3+2] cycloadditions reactions [49]. 

## 2. Results

The choice of the cyclopropenimine base was guided by its high basic strength, pK_BH_+ = 26.9, which is similar to that of the Schwesinger base P1-t-Bu [50]. The high basicity of cyclopropenimines is due to the aromatic cyclopropenium ion, which is formed upon protonation at the imino nitrogen [51]. In addition, at variance with phosphazene bases, the cyclopropenimine scaffold can be easily decorated with chiral moieties providing an easy entry to chiral catalysts. 

The chiral enantiopure cyclopropenimine **I** is easily accessible on the multigram scale in a straightforward manner, see Materials and Methods section, alternatively, base **I** is also commercially available. 

**Figure 1:**
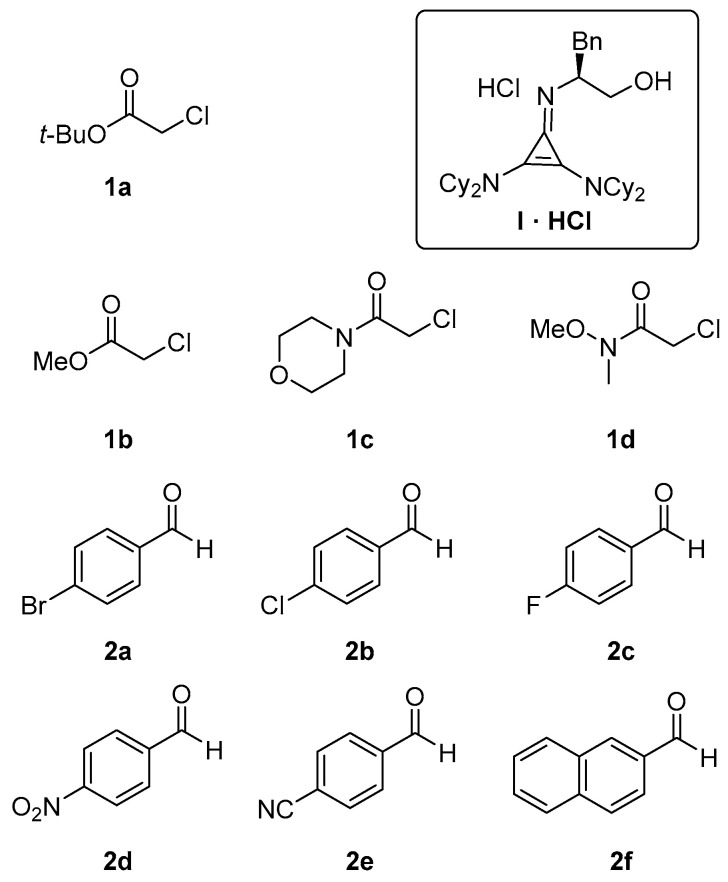
Structures of pronucleophiles (**1a**–**1d**), aldehydes (**2a**–**2f**) and catalyst (**I**) used in the present study.

At the outset of our study, we carried out a preliminary investigation of the Darzens reaction between *t*-butyl chloroacetate (**1a**) and 4-bromobenzaldehyde (**2a**), Figure 1, seeking the best solvent system. This screening was performed by using a stoichiometric amount of cyclopropenimine free base **I** or its hydrochloride salt **I·HCl** [47], with respect to the α-haloester and the aldehyde, Table 1. When using **I·HCl**, the reactions were carried out in the presence of concentrated aqueous KOH to ensure the generation of the free base **I**. For this screening, the reactions were carried out on 0.25 mmol of aldehyde using a **1a**/**2a**/**I** or **1a**/**2a**/**I·HCl** molar ratio of 1.5:1:1.5 in 1 mL of solvent at 25 °C. The solvents considered were: dry CH_3_CN, dry ethyl acetate, which has often been employed in reactions involving cyclopropenimine bases [52], CH_2_Cl_2_ or toluene.

The use of CH_3_CN resulted in 44% conversion after 16 h and 34% isolated yield of epoxide **3aa**, Table 1 entry 1, while the reaction performed in dry ethyl acetate gave no conversion to the product after 24 h. Using a stoichiometric amount of the hydrochloride salt **I·HCl** instead of the free base in solvents such as DCM or toluene in the presence of concentrated, aqueous KOH gave low conversions and yields. However, under these biphasic conditions, lowering the amount of **I·HCl** to 20 mol% still gave conversion to the product similar to that achieved using a stoichiometric amount of **I·HCl**, Table 1, entry 4 vs. entry 3. Considering that under the same conditions but in the absence of a catalyst, no reactions occurred, the above observation pinpoints that free base **I** generated by in situ deprotonation of the hydrochloride salt is catalytically active and the hydroxide anion promoted Darzens condensation is negligible. 

This observation prompted us to explore different reaction conditions in order to optimize the conversion, considering systems comprising low polarity solvents, two equivalents of alkali metal carbonates as bases and a 20 mol% of **I·HCl** as catalyst. When the reaction was performed in acetonitrile, using Cs_2_CO_3_ the product was obtained in low yield regardless of the presence of catalyst, Table 2 entry 1 vs. entry 2. 

On the contrary, by using potassium carbonate, the reaction proceeds only in the presence of **I·HCl** affording the product with 49% conversion and 36% isolated yield, Table 2, entries 3 and 4. 

Increasing the amount of potassium carbonate to four equivalents and increasing the amount of **I·HCl** to 30 mol% yielded almost quantitative conversion to the product with a 67% isolated yield because of product instability under chromatographic conditions, Table 2 entry 6 vs. entry 5; without the addition of **I·HCl** the reaction did not proceed despite the increased amount of potassium carbonate. Under these conditions, the condensation of methyl chloroacetate was less efficient than that of *t*-butyl chloroacetate, Table 2, entry 7 vs. entry 4.

Other solvents such as THF or toluene, in combination with K_2_CO_3_ or Cs_2_CO_3_ proved unsuitable for this reaction since no conversion could be observed in 16 h, Table 2, entries 8–9. Organic bases such as pyridine, *N*,*N*-Diisopropylethylamine (DIPEA) and *N*,*N*,*N*′,*N*′-1,8-bis(dimethylamino)naphthalene (Proton Sponge) used in dichlorometane, or acetonitrile were also considered in the screening but did not provide measurable conversion to the product, Table 2, entries 10–14. 

This survey allowed us to pinpoint that the best conditions require the use of acetonitrile as a solvent in the presence of 30 mol% of **I·HCl** and the use of 4 molar equivalents of potassium carbonate. Under these optimized conditions the performance of **I·HCl** (30 mol %) was assessed against various aromatic aldehydes and pronucleophiles, Figure 1 and Table 3.

The reactions proceed smoothly, generally scoring high conversion within 16 h. With *p*-chlorobenzaldehyde (**2b**) and *p*-fluorobenzaldehyde (**2c**) the epoxides derived from the pronucleophile **1a** were obtained with 65% and 32% isolated yield, respectively. With the 4-cyanobenzaldehyde (**2e**), Table 3 entry 4, the reaction displayed a full conversion and the epoxide **3ae** was obtained with 78% yield and 1/0.7 *cis*/*trans* ratio. Also, 2-naphthaldehyde **2f** displayed good reactivity under these conditions.

Aldehyde **2e** was then selected to test the reactivity of different pronucleofiles, including *N*-(Chloroacetyl)morpholine **1c**, Table 3 entry 6, and the Weinreb amide of chloroacetic acid **1d**, Table 3 entry 7. In the first case the product was obtained with low conversion and yield, while in the latter, the product was obtained with quantitative conversion enabling the preparation of epoxide **3de** with an excellent isolated yield. In particular, the preparation of Weinreb amides of aryl glycidic acids was achieved so far only by catalytic oxidation of the cinnamic acid amides [53,54], amidation of the free acids which are known to be unstable, or by reaction of sulfur yilides obtained from diazo acetamides [55]. 

## 3. Discussion

Our preliminary experiments using *t*-butyl-chloroacetate (**1a**) as pronucleophile and *p*-bromobenzaldehyde (**2a**) as the carbonyl component displayed that the basicity of cyclopropenimine **I** was sufficient to carry out smooth stoichiometric deprotonation of the α-haloester in low polarity solvents such as acetonitrile, dichloromethane and toluene. The cyclopropenimine **I** can be used as a free base or, alternatively, the hydrochloride salt **I·HCl** can be also used provided that a sacrificial base such as KOH is introduced in the reaction system as a concentrated aqueous solution. The use of **I·HCl** represents an advantage since it has been reported that free base **I** is unstable and rearranges to the corresponding oxazoline while **I·HCl** is an essentially indefinitely stable compound [52]. Control experiments performed in order to assess any participation of KOH in the reaction displayed that under these heterogeneous conditions the background Darzens condensation not involving **I** was negligible, in line with literature reports that highlight the necessity of phase transfer catalysis to achieve this transformation [32,56,57,58,59]. Moreover, by lowering the amount of **I·HCl** to substechiometric, we could still observe measurable conversion to the products, pinpointing that base **I** can be used to devise a catalytic approach to this transformation. To this end, using 30 mol% of **I·HCl**, we addressed a sacrificial base screening primarily exploring heterogeneous systems in which the base was introduced as a solid phase. Alkali metal carbonates such as K_2_CO_3_ or Cs_2_CO_3_ proved to be effective only in combination with acetonitrile as a solvent; however, the reactions performed using Cs_2_CO_3_ suffered a significant contribution of the background, uncatalysed, reaction, likely because of the higher solubility of this salt in acetonitrile due to the softer nature of the Cs^+^ cation respect to K^+^ [60]. Under optimized conditions, the Darzens reaction involving *t*-butyl-chloroacetate (**1a**) and *p*-bromobenzaldehyde (**2a**) required 4 molar equivalents of K_2_CO_3_ and a 30% of **I·HCl** with respect to the substrates. The conversion of the reagents, assessed after 16 h, was 93% and the product could be isolated in 67% yield. Control experiments confirmed that in the absence of **I·HCl**, the transformation is ineffective also under these conditions. In all of these reactions the diastereoisomeric products are formed with only limited selectivity for the *cis* isomer, both the *cis* and the *trans* isomers were however found to be racemic, despite the enantiopure nature of base **I**, see the Supplementary Materials. The *cis* selectivity observed, despite limited, shares some similarities with the general outcome of the Darzens reactions carried out under PCT conditions [61]. We speculate that, by analogy to those conditions, the *cis* selectivity might be partly accounted for considering the steric bulk of base **I**. Indeed, under PCT, the most favorable ion pair formed upon nucleophilic addition of the ester enolate to the aldehyde is the one that better accommodates the sterically demanding quaternary ammonium ions; this intermediate leads to the *cis* epoxy ester [61].

**Figure 2:**
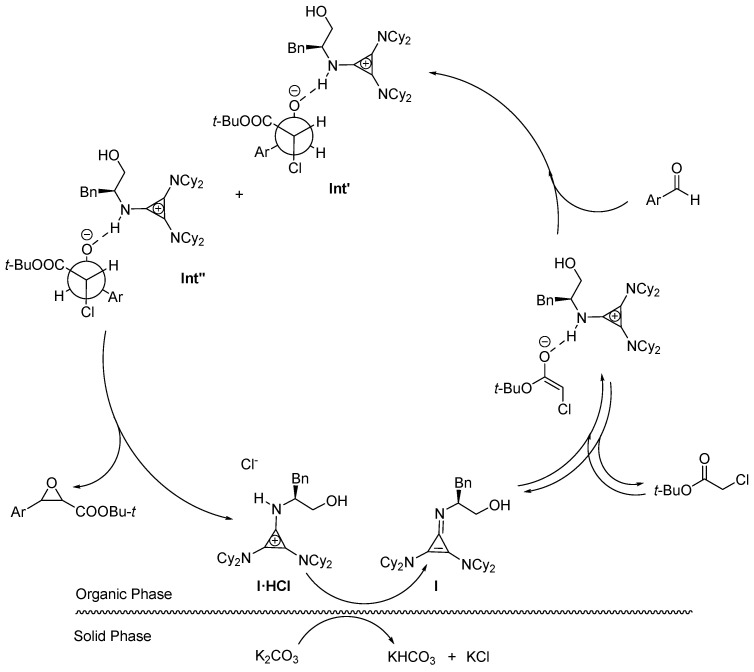
Proposed catalytic cycle involving **I·HCl**.

A plausible mechanism for the reaction explaining the catalytic role of base **I** can therefore be sketched based on our observations and the available information on the base-promoted Darzens reactions [32,56,57,58,59,61], Figure 2. We consider that **I·HCl** will be first deprotonated by the sacrificial heterogeneous base generating the free base in the organic phase. Base **I** will enter a deprotonation equilibrium leading to the ester enolate that will provide nucleophilic addition to the aldehyde carbonyl group. As mentioned above, the little *cis*-diastereoelectivity observed in our experiments led us to assume the almost equimolar formation of the two diastereomeric haloidrin anion intermediates **Int′** and **Int″**, with a slight preference for **Int′** in which the protonated base can occupy the least hindered side of the intermediate. Finally, by epoxide ring closure, **I·HCl** is reformed closing the catalytic cycle.

Other organic bases such as DIPEA and Proton Sponge were used in order to extend our study to homogeneous systems. Not surprisingly, in the presence of these species, no conversion to the product could be observed because of their weaker basicity with respect to cyclopropenimine I, making it impossible to achieve significant concentrations of free base I starting from the hydrochloride salt.

The catalytic system developed proved to be effective in promoting the Darzens condensations of aromatic aldehydes with *t*-butyl chloroacetate achieving good to excellent conversions of the reagents. Other pronucleophiles such as *N*-(Chloroacetyl)morpholine and the Weinreb amide of chloroacetic acid were also tested providing the condensation products in moderate to good yields. 

## 4. Materials and Methods

### 4.1. General Information

Unless otherwise noted, all reactions were performed in oven-dried or flame-dried glassware. Air-sensitive reagents and solutions were transferred via a syringe and were introduced to the apparatus through rubber septa. All reagents were purchased from Sigma-Aldrich srl (Milan, Italy). or Alfa Aesar GmbH (Karlsruhe, Germany) and used as received. All solvents were purchased from Sigma-Aldrich Co. LLC or Alfa Aesar GmbH. Dry dichloromethane, dry acetonitrile, dry ethyl acetate, dry THF, toluene (ACS grade) were used as received. Solvents for chromatography and filtration including ethyl acetate, dichloromethane, petroleum ether and methanol were used as received; hexane and 2-propanol were HPLC grade. Analytical thin layer chromatography (TLC) was performed on silica gel 60 F254 pre-coated plates with visualization under short-wavelength UV light. Additionally, spots were visualized by dipping the plates with potassium permanganate (aqueous H_2_SO_4_ solution of potassium permanganate) and ninhydrin reagent (n-butanol solution of ninhydrin and acetic acid) followed by heating. Flash column chromatography was performed using Biotage^®^ SNAP Cartridge KP-Sil 10 g, Biotage apparatus and the indicated solvent mixtures. Analytical chiral HPLC analyses were carried out using the indicated columns, solvents and conditions. 

Proton NMR spectra were recorded at 400 MHz (Bruker 400 MHz). Carbon NMR spectra were recorded at 100 MHz (Bruker 400 MHz). The proton chemical shifts were referenced to the residual non deuterated solvent (δ = 7.26 for CDCl_3_; δ = 2.49 for DMSO-*d*_6_). Chemical shifts (δ) are reported in parts per million (ppm), and multiplicities are indicated as s (singlet), d (doublet), t (triplet), q (quartet), dd (double doublet), m (multiplet), and b (broad). Coupling constants, J, are quoted in Hertz. ^1^H and ^13^C NMR assignments were supported by 2D experiments (gCOSY, gHSQC, ROESY experiments). 

ESI-mass spectra were recorded on AcquityTM Ultra Performance LC apparatus and are reported in the form of (*m*/*z*). LC runs were performed using an Acquity UPLC CSH C18 column (50 mm × 2.1 mm i.d. 1.7 μm particle size) at 40 °C; solvents: A = 0.1% *v/v* solution of HCOOH in water B = 0.1% *v/v* solution of HCOOH in acetonitrile; gradient: from 3% to 99.9% of solvent B; flow rate: 1 mL/min; acquisition stop time: 2.0 min.

### 4.2. Preparation of Catalyst ***I·HCl***

Catalyst I was prepared according to reported procedures, and its spectral data perfectly matched those reported in the literature [40,43].

Dicyclohexylamine (33.5 mL, 168.66 mmol) was slowly added to a solution of tetrachlorocyclopropene (5 g, 28.11 mmol) in CH_2_Cl_2_ (280 mL) in a 1L round bottom flask. A white precipitate was formed. The reaction mixture was stirred for 4 h at 25 °C. Next, (*S*)-2-amino-3-phenylpropan-1-ol (4.67 g, 30.92 mmol) was added in one portion and the reaction mixture was stirred for an additional 10 h. The crude reaction mixture was filtered through a celite plug, then washed with 1.0 M HCl (3 × 130 mL), dried over anhydrous Na_2_SO_4_, filtered and concentrated under vacuum to yield pure cyclopropenimine hydrochloride salt **I·HCl** (16.3 g, >99% yield) as yellow solid.

### 4.3. Preparation of Compound ***3*** in the Presence of Catalyst ***I·HCl***

To a solution of aldehyde **2** (0.25 mmol, 1.0 equiv.) and *α*-halo carbonyl compound **1** (0.375 mmol, 1.5 equiv.) in anhydrous acetonitrile (1 mL), catalyst **I·HCl** (43 mg, 0.075 mmol) and K_2_CO_3_ (138 mg, 1.0 mmol) were added at 25 °C. The resulting mixture was stirred at 25 °C for 16 h. Then, a saturated aqueous solution of ammonium chloride (1 mL) was added. The resulting mixture was extracted with CH_2_Cl_2_ (3 × 2 mL). The organic layers were combined, dried over Na_2_SO_4_ anhydrous, filtered and concentrated in vacuum to yield a crude compound. The crude compounds were purified by silica gel flash chromatography (90/10 cyclohexane/ethyl acetate) to yield compound **3**, spectroscopic data match those reported in the literature [32,61,62].

## 5. Conclusions

In summary, in this work, we explored the feasibility of Darzens condensation reactions between α-chloroesters and substituted aromatic aldehydes promoted by a series of organic bases. Low basicity amines such as DIPEA and pyridine used in a stoichiometric amount proved to be ineffective but also the higher basicity Proton Sponge did not allow to observe measurable conversion to the products. On the contrary, the use of cyclopropenimine superbase **I** allowed us to achieve smooth conversion of the reagents. Furthermore, the cyclopropenimine superbase I was proved to be active even at substoichiometric levels, in the presence of excess K_2_CO_3_ as a sacrificial base, thus enabling the set-up of a heterogeneous catalytic system for Darzens Reactions. Under our optimised conditions, the reaction of *α*-haloesters and amides with a series of aromatic aldehydes afforded *α*,*β*-epoxyesters and *α*,*β*-epoxyamides in high conversions and acceptable to excellent yields. To the best of our knowledge, this is the first report in which a cyclopropenimine superbase is used either stoichiometrically or catalytically for this kind of transformations. The low nucleophilicity of these bases makes this method a potentially valuable alternative to other base promoted/catalysed Darzens reactions. A plausible mechanistic hypothesis is provided to account for the limited diastereoselectivity of the reactions that displayed only a slight preference for the formation of the *cis*-epoxide. We consider that the steric bulk of cyclopropenimine **I** is primarily responsible for the limited, but consistent throughout the study, selectivity for the cis-epoxide products in analogy with the Darzens reaction performed under PTC conditions. However, since the cyclo-propenimine scaffold is amenable to decoration with various fragments at the imino nitrogen, we envision that substituents capable of providing further interactions with the aldehyde carbonyl group will possibly improve the strereoselectivity of our method.

## Figures and Tables

**Table 1:** Darzens reaction of *t*-butyl chloroacetate (**1a**) and 4-bromobenzaldehyde (**2a**) in the presence of stoichiometric cyclopropenimine **I** or its hydrochloride salt **I HCl** ^1^.

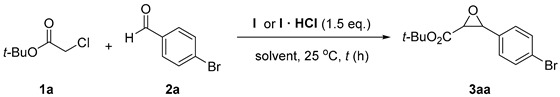					
Entry	Solvent	Time (hours) ^2^	Conv. (%) ^3,4^	Yield (%) ^3,5^	(*cis*/*trans*) ^3,4,6^
1 ^7^	dry CH_3_CN	16	44	34	1/0.85
2 ^7^	dry EtOAc	24	-	-	-
3	CH_2_Cl_2_/KOH aq. 50% ^8^	16	33	21	1/0.6 ^9^
4 ^10^	CH_2_Cl_2_/KOH aq. 50% ^8^	16	17	-	-
5	toluene/KOH aq. 50% ^8^	16	28	17	1/0.8 ^9^

^1^ Unless otherwise stated, all Darzens reactions were carried out on a 0.25 mmol scale using 1.5:1:1.5 **1a**/**2a**/**I** molar ratio in 1 mL of solvent at 25 °C. ^2^ Reaction time. ^3^ Determined by ^1^H NMR analysis on the crude reaction mixture. ^4^ Average of two experiments. ^5^ Yield of isolated product after column chromatography. ^6^ The *cis* and *trans* epoxides formed under these conditions are racemic, the analyses were performed by chiral HPLC. ^7^ Free base of catalyst **I** was used. ^8^ 2.0 eq. of KOH aq. 50% were used. ^9^ The *cis*/*trans* ratios were determined by ^1^H NMR analysis performed on the purified compound **3aa**. ^10^ 20 mol % of catalyst **I·HCl** were used.

**Table 2:** Development of Darzens reaction in the presence of catalytic loading of **I·HCl** ^1^.

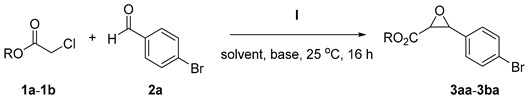							
Entry	R	Solvent	Base	I·HCl (mol %)	Conv. (%) ^2,3^	Yield (%) ^3,4^	*cis*/*trans* ^2,5^
1 ^6^	*t*-Bu	CH_3_CN	Cs_2_CO_3_	-	18	-	-
2	*t*-Bu	CH_3_CN	Cs_2_CO_3_	20	14	-	-
3	*t*-Bu	CH_3_CN	K_2_CO_3_	-	-	-	-
4	*t*-Bu	CH_3_CN	K_2_CO_3_	20	49	36	1/0.7
5 ^7^	*t*-Bu	CH_3_CN	K_2_CO_3_	20	85	60	1/0.7
6 ^7^	*t*-Bu	CH_3_CN	K_2_CO_3_	30	93	67	1/0.7
7	Me	CH_3_CN	K_2_CO_3_	20	16	-	-
8	*t*-Bu	THF	K_2_CO_3_	20	-	-	-
9	*t*-Bu	Toluene	Cs_2_CO_3_	20	-	-	-
10	*t*-Bu	CH_2_Cl_2_	Pyridine	20	-	-	-
11	Me	CH_3_CN	DIPEA	20	-	-	-
12	Me	CH_3_CN	Proton Sponge	20	-	-	-
13	Me	CH_2_Cl_2_	DIPEA	20	-	-	-
14	Me	CH_2_Cl_2_	Proton Sponge	20	-	-	-

^1^ Unless otherwise stated, all Darzens reactions were carried out on 0.25 mmol of the aldehyde **2a**, with a 1.5:1:2 **1**/**2a**/base molar ratio in 1 mL of solvent at 25 °C. The reaction without catalyst **I·HCl** did not provide compound **3**. ^2^ Determined by ^1^H NMR analysis of the crude reaction mixture. ^3^ Average of two experiments. ^4^ Yield of isolated product after column chromatography. ^5^ The cis and trans epoxides formed under these conditions are racemic, the analyses were performed by chiral HPLC. ^6^ Reaction performed without **I·HCl**. ^7^ 4.0 eq. of K_2_CO_3_ were used.

**Table 3:** Catalytic Darzens reactions of chloroacetate esters and amides with aromatic aldehydes in the presence of catalyst **I·HCl** ^1^.

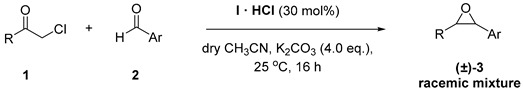					
Entry	Pronucleophile	Aldehyde	Conv.(%) ^2^	Yield (%) ^3^	*cis*/*trans* ^2^
1	**1a**	**2b**	88	65	1/0.7
2	**1a**	**2c**	66	32	1/0.9
3	**1a**	**2d**	87	47	1/0.7
4	**1a**	**2e**	>99	78	1/0.7
5	**1a**	**2f**	80	41	1/0.6
6	**1c**	**2e**	48	32	1/0.9
7	**1d**	**2e**	>99	86	1/0.75

^1^ Unless otherwise stated, all Darzens reactions were carried out on a 0.25 mmol of the aldehyde with a **1**/**2**/K_2_CO_3_ molar ratio of 1.5:1:4 in 1 mL of dry MeCN at 25 °C. ^2^ Determined by ^1^H NMR analyses of the crude reaction mixture. ^3^ The yields are those of the isolated products with an average of 2 experiments.

## Data Availability

The original data not present in the article main text are reported in the Supplementary Materials.

## Supplementary Materials

The following supporting information can be downloaded at: https://www.mdpi.com/article/10.3390/molecules29184350/s1, characterization data for catalyst **I·HCl**, characterization data for compounds **3ab**, **3ac**, **3ad**, **3ae**, **3af**, **3ce**, **3de**. References are cited in [32,61,62].
