# Supplementary material for: Development of Organocatalytic Darzens Reactions Exploiting the Cyclopropenimine Superbase
Source: Molecules. 2024 Sep 13;29(18):4350. doi: 10.3390/molecules29184350 (PMC11434499; doi:10.3390/molecules29184350)
Supplement: Supplementary file 1 [file molecules-29-04350-s001.zip › molecules-3158380-supplementary.pdf]

Development of Organocatalytic Darzens Reactions Exploiting the Cyclopropenimine Superbase

Carmine Lops †, Lucia Pasquato * and Paolo Pengo *

Department of Chemical and Pharmaceutical Sciences, University of Trieste, Via Licio Giorgieri 1, 34127 Trieste, Italy; carmine.lops@hotmail.com

* Correspondence: lpasquato@units.it (L.P.); ppengo@units.it (P.P.)

† Current address: Aptuit, Via A. Fleming 4, 37135 Verona, Italy.

SUPPLEMENTARY MATERIALS

Table of content

Analytical and spectroscopic data of catalyst **I·HCl**	Page S1
Analytical and spectroscopic data for **3aa**	Page S2
Analytical and spectroscopic data for **3ab**	Page S3
Analytical and spectroscopic data for **3ac**	Page S4
Analytical and spectroscopic data for **3ad**	Page S5
Analytical and spectroscopic data for **3ae**	Page S7
Analytical and spectroscopic data for **3af**	Page S9
Analytical and spectroscopic data for **3ce**	Page S10
Analytical and spectroscopic data for **3de**	Page S12
^{1}H NMR, ^{13}C NMR of Representative Compounds	Page S14
References	Page S23

Analytical and spectroscopic data of catalyst **I·HCl**

^{1}H NMR (400 M MHz, $CDCl_3$): δ 7.78 (d, *J* = 9.5 Hz, 1H, N*H*), 7.25-7.12 (m, 5H, Ar*H*), 5.2 (bs, 1H, -O*H*), 4.05-3.78 (m, 3H, NC*H*BnC*H*$_2$OH), 3.27 (ddd, *J* = 11.9, 8.4, 3.8 Hz, 4H, NCy*H*), 3.16-3.00 (m, 2H, -C*H*$_2$Ph), 1.98-1.00 (m, 40H, Cy*H*).

^{13}C NMR (100 MHz, $CDCl_3$): δ 138.43 (1C, C=N), 129.52 (2C, Ar), 128.24 (2C, Ar), 126.33 (1C, Ar), 117.41 (Cq), 114.83 (Cq), 64.08 (1C, NCHBn*C*H$_2$OH), 61.9 (1C, N*C*HBnCH$_2$OH), 59.43 (4C, -NCy), 38.61 (1C, -*C*H$_2$Ph), 32.41 (4C, Cy), 32.24 (4C, Cy), 25.73 (4C, Cy), 25.67 (4C, Cy), 24.68 (4C, Cy).

MS (ESI, 5600eV): Calcd.:[M+H$^+$]: 546.85; Found: [M+H$^+$]: 546.28.

Ultra Performance LC analysis:

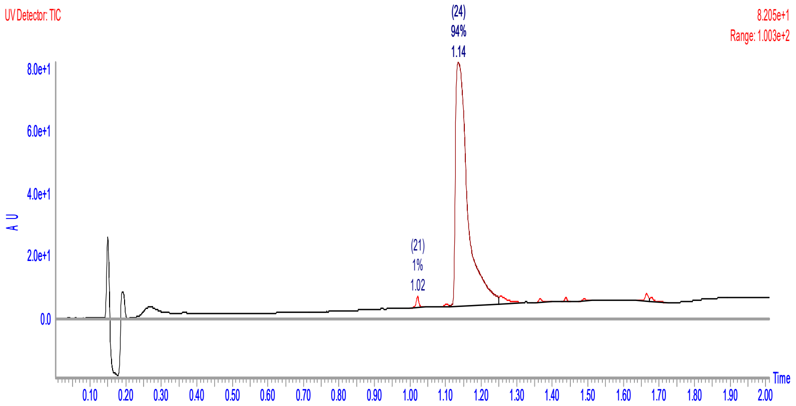

Rt (minutes)	Area (%)
1.14	94

Analytical and spectroscopic data for **3aa**

(67% yield, *cis*/*trans*= 1/0.7, white solid):

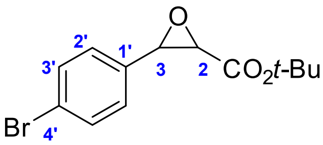

Trans ^{1}H NMR (400 M MHz, $CDCl_3$): δ 7.5 (d, J = 8.0 Hz, 2H, H-3'), 7.18 (d, J = 8.3 Hz, 2H, H-2'), 3.99 (m, 1H, H-3), 3.36 (d, J = 1.5 Hz, 1H, H-2), 1.54 (s, 9H). ^{13}C NMR (100 MHz, $CDCl_3$): δ 166.86 (1C, C=O), 134.44 (1C, C-1'), 131.78 (2C, C-3′), 127.5 (2C, C-2'), 122.86 (1C, C-4'), 82.9 (1C, *C*H(CH_3)$_3$), 57.39 (1C, C-2), 57.01 (1C, C-3), 28.00 (3C, CH(*C*H_3)$_3$).
Cis ^{1}H NMR (400 M MHz, $CDCl_3$): δ 7.48 (d, J = 8.0 Hz, 2H, H-3'), 7.31 (d, J = 8.5 Hz, 2H, H-2'), 4.17 (d, J = 4.5 Hz, 1H, H-3), 3.72 (d, J = 4.5 Hz, 1H, H-2), 1.23 (s, 9H).[1] ^{13}C NMR (100 MHz, $CDCl_3$): δ 165.45 (1C, C=O), 132.32 (1C, C-1'), 131.04 (2C, C-3′), 128.5 (2C, C-2'), 122.3 (1C, C-4'), 82.63 (1C, *C*H(CH_3)$_3$), 56.56 (1C, C-3), 55.89 (1C, C-2), 27.74 (3C, CH(*C*H_3)$_3$).
MS (ESI, 5600eV): Calcd:[M+H$^+$]: 258.08; Found: [M+H$^+$]: 258.8
R$_f$: 0.35 (18/2 Cyclohexane/EtOAc).

Chiral HPLC: Chiralpak IA (25 × 0.46 cm), 5 µm, *n*-hexane/EtOH = 85/15, 1 ml/min, 220 nm.

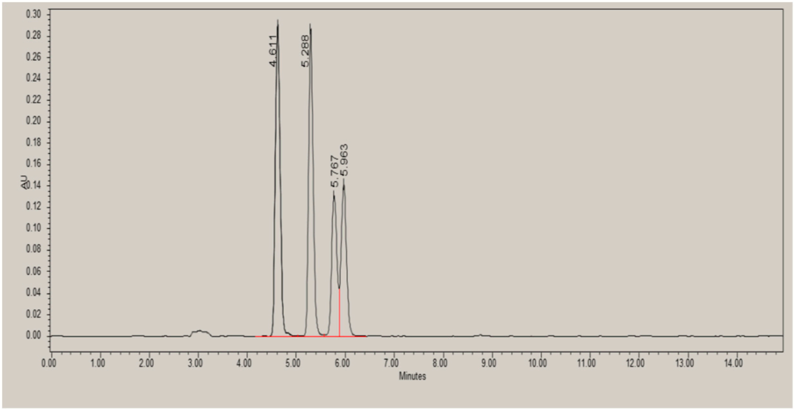

Rt (minutes)	Area (%)
4.6	33.8
5.2	31.2
5.7	16.7
5.9	18.3

Analytical and spectroscopic data for **3ab**

(65% yield, *cis*/*trans* = 1/0.7, white solid):

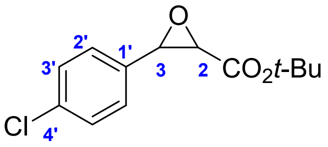

Trans ^{1}H NMR (400 M MHz, CDCl$_3$): δ 7.43-7.2 (m, 4H), 4.02 (d, *J* = 1.5 Hz, 1H, H-3), 3.38 (d, *J* = 1.8 Hz, 1H, H-2), 1.57 (s, 9H, *t*-Bu).[2] ^{13}C NMR (100 MHz, CDCl$_3$): δ 166.91 (1C, C=O), 134.75 (1C, Cq), 133.9 (1C, Cq) 128.84 (2C, C-3′), 127.22 (2C, C-2'), 82.9 (1C, *C*(CH$_3$)$_3$), 57.44 (1C, C-2), 56.97 (1C, C-3), 28.00 (3C, CH(*C*H$_3$)$_3$).
Cis ^{1}H NMR (400 M MHz, CDCl$_3$): δ 7.43-7.2 (m, 4H), 4.21 (d, *J* = 4.8 Hz, 1H, H-3), 3.74 (d, *J* = 4.8 Hz, 1H, H-2), 1.24 (s, 9H, *t*-Bu).[2] ^{13}C NMR (100 MHz, CDCl$_3$): δ 165.48 (1C, C=O), 134.17 (1C, Cq), 131.78 (1C, Cq) 128.19 (2C, C-3′), 128.1 (2C, C-2'), 82.61 (1C, *C*(CH$_3$)$_3$), 56.51 (1C, C-2), 55.96 (1C, C-3), 27.73 (3C, CH(*C*H$_3$)$_3$).
MS (ESI, 5600eV): Calcd.: [M+H$^+$]: 255.71; Found: [M+H$^+$]: 255.02.
R$_f$: 0.36 (18/2 Cyclohexane/EtOAc).

Ultra Performance LC analysis:

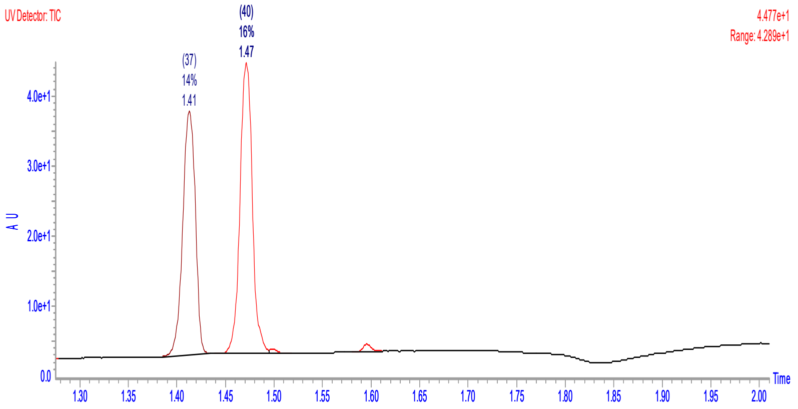

Rt (minutes)	Area (%)
1.41	14
1.47	16

Chiral HPLC:_Chiralpak IA (25 × 0.46 cm), 5 µm, *n*-hexane/EtOH = 90/10, 1 ml/min, 220 nm.

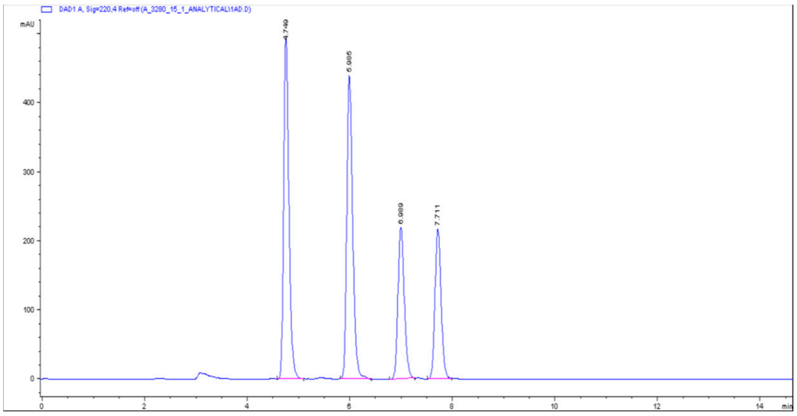

Rt (minutes)	Area (%)
4.7	32.9
5.9	32.7
7.0	17.6
7.7	16.8

Analytical and spectroscopic data for **3ac**

(32% yield, *cis*/*trans* = 1/0.9, white solid):

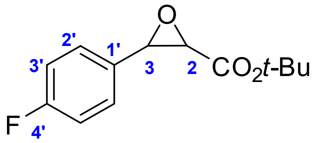

Trans ^{1}H NMR (400 M MHz, $CDCl_3$): δ 7.31–7.22 (m, 2H, H-2'), 7.09-7,01 (m, 2H, H-3'), 4.01 (d, *J* = 1.3 Hz, 1H, H-3), 3.37 (d, *J* = 1.5 Hz, 1H, H-2), 1.52 (s, 9H). ^{13}C NMR (100 MHz, $CDCl_3$): δ 167.03 (1C, C=O), 163.08 (d, *J* = 245 Hz, C-4'), 131.1 (d, *J* = 2 Hz, C-1'), 127.6 (d, *J* = 8 Hz, C-2'), 115.65 (d, *J* = 21 Hz, C-3'), 82.82 (1C, *C*(CH_3)$_3$), 57.41 (1C, C-2), 57.05 (1C, C-3), 28.00 (3C, CH(*C*H_3)$_3$).**Error! Bookmark not defined.**

Cis ^{1}H NMR (400 M MHz, $CDCl_3$): δ 7.44–7.36 (m, 2H, H-2'), 7.09-7,01 (m, 2H, H-3'), 4.2 (d, *J* = 4.5 Hz, 1H, H-3), 3.7 (d, *J* = 4.5 Hz, 1H, H-2), 1.22 (s, 9H). ^{13}C NMR (100 MHz, $CDCl_3$): δ 165.6 (1C, C=O), 162.7 (d, *J* = 245 Hz, C-4'), 129.01 (d, *J* = 3 Hz, C-1'), 128.5 (d, *J*

= 8 Hz, C-2'), 114.89 (d, *J* = 22 Hz, C-3'), 82.51 (1C, *C*(CH_3)$_3$), 56.53 (1C, C-2), 55.98 (1C, C-3), 27.71 (3C, CH(CH_3)$_3$).

MS (ESI, 5600eV): Calcd.:[M+H$^+$]: 239.25; Found: [M+H$^+$]: 238.95

R$_f$: 0.32 (18/2 Cyclohexane/EtOAc).

Ultra Performance LC analysis:

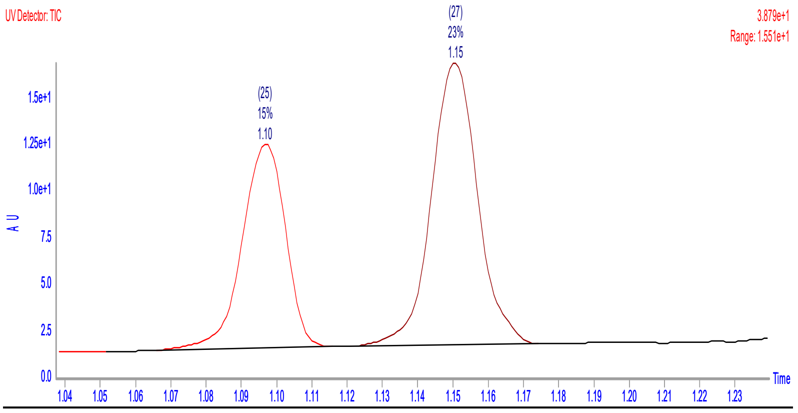

Rt (min)	Area (%)
1.1	15
1.15	23

Chiral HPLC: Chiralpak AY-H (25 × 0.46 cm), 5 µm, *n*-hexane/EtOH = 85/15, 1 ml/min, 220 nm.

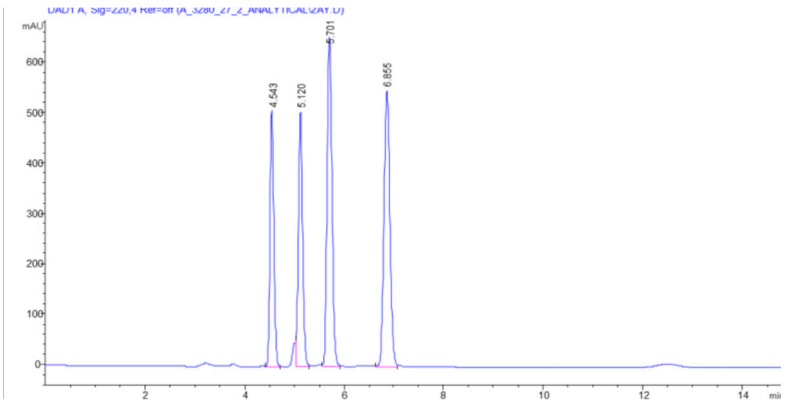

Rt (minutes)	Area (%)
4.5	18.6
5.1	20.5
5.7	29.9
6.8	31.0

Analytical and spectroscopic data for **3ad**

(47% yield, *cis*/*trans* = 1/0.7, white solid):

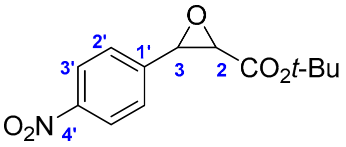

Trans ^{1}H NMR (400 M MHz, $CDCl_3$): δ 8.27–8.17 (m, 2H, H-3'), 7.50 (d, J = 8.5 Hz, 2H, H-2'), 4.13 (d, J = 1.3 Hz, 1H, H-3), 3.39 (d, J = 1.3 Hz, 1H, H-2), 1.54 (s, 9H).

Cis ^{1}H NMR (400 M MHz, $CDCl_3$): δ 8.27–8.17 (m, 2H, H-3'), 7.62 (d, J = 8.5 Hz, 2H, H-2'), 4.3 (d, J = 4.5 Hz, 1H, H-3), 3.8 (d, J = 4.5 Hz, 1H, H-2), 1.22 (s, 9H).[3]

^{13}C NMR (100 MHz, $CDCl_3$): δ 166.28 (1C, C=O), 164.91 (1C, C=O), 142.64 (1C), 140.48 (1C), 130.47 (1C), 127.87 (1C, C-2', *cis*), 126.7 (1C, C-2', *trans*), 124.31 (1C), 123.89 (1C, C-3', *trans*), 123.11 (1C, C-3', *cis*), 83.36 (1C), 83.04 (1C), 57.7 (1C, *trans*), 56.42 (1C, *trans*), 56.25 (1C, *cis*), 55.96 (1C, *cis*), 27.98 (3C, CH(CH_3)$_3$, *trans*), 27.75 (3C, CH(CH_3)$_3$, *cis*).[3]

MS (ESI, 5600eV): Calcd.: [M+H$^+$]: 266.26; Found: [M+H$^+$]: 266.04.

R$_f$: 0.29 (18/2 Cyclohexane/EtOAc).

Ultra Performance LC analysis:

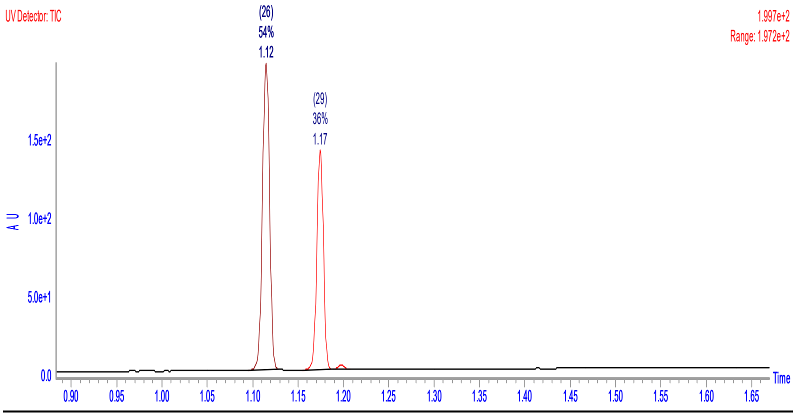

Rt (min)	Area (%)
1.12	54
1.17	36

Chiral HPLC: Chiralpak IA (25 × 0.46 cm), 5 µm, *n*-hexane/EtOH = 70/30, 1 ml/min, 220 nm.

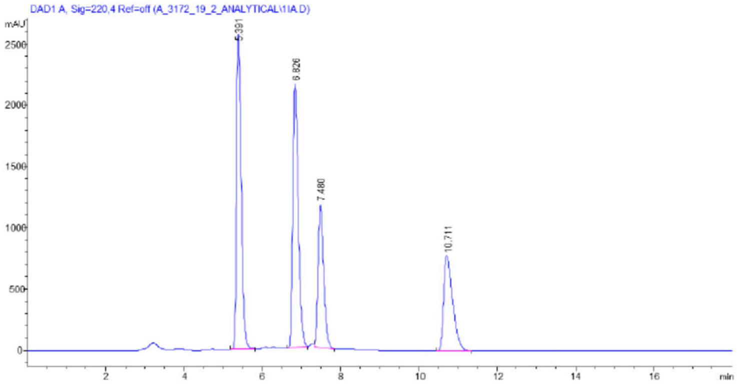

Rt (minutes)	Area (%)
5.3	32
6.8	32.3
7.4	17.2
10.7	18.5

Analytical and spectroscopic data for **3ae**

(78% yield, *cis*/*trans* = 1/0.7, white solid):

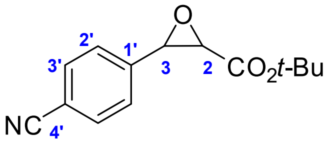

Trans ^{1}H NMR (400 M MHz, $CDCl_3$): δ 7.71–7.63 (m, 2H, H-3'), 7.42 (d, *J* = 8.0 Hz, 2H, H-2'), 4.09 (d, *J* = 1.3 Hz, 1H, H-3), 3.37 (d, *J* = 1.5 Hz, 1H, H-2), 1.56 (s, 9H).[2]

Cis ^{1}H NMR (400 M MHz, $CDCl_3$): δ 7.71–7.63 (m, 2H, H-3'), 7.56 (d, *J* = 8.0 Hz, 2H, H-2'), 4.25 (d, *J* = 4.8 Hz, 1H, H-3), 3.78 (d, *J* = 4.8 Hz, 1H, H-2), 1.21 (s, 9H).[2]

^{13}C NMR (100 MHz, $CDCl_3$): δ 166.38 (1C, C=O), 165.00 (1C, C=O), 140.74 (1C), 138.58 (1C), 132.43 (2C), 131.69 (2C), 127.65 (2C, C-2', *cis*), 126.53 (2C, C-2', *trans*), 118.52 (1C), 118.36 (1C), 112.71 (1C), 112.19 (1C), 83.28 (1C), 82.96 (1C), 57.65 (1C, C-3, *trans*), 56.61 (1C, C-2, *trans*), 56.34 (1C, C-3, *cis*), 55.93 (1C, C-2, *cis*), 27.98 (3C, $CH(CH_3)_3$, *trans*), 27.71 (3C, $CH(CH_3)_3$, *cis*).

MS (ESI, 5600eV): Calcd.: [M+H$^+$]: 246.27; Found: [M+H$^+$]: 245.99

R*f*: 0.35 (18/2 Cyclohexane/EtOAc).

Ultra Performance LC analysis:

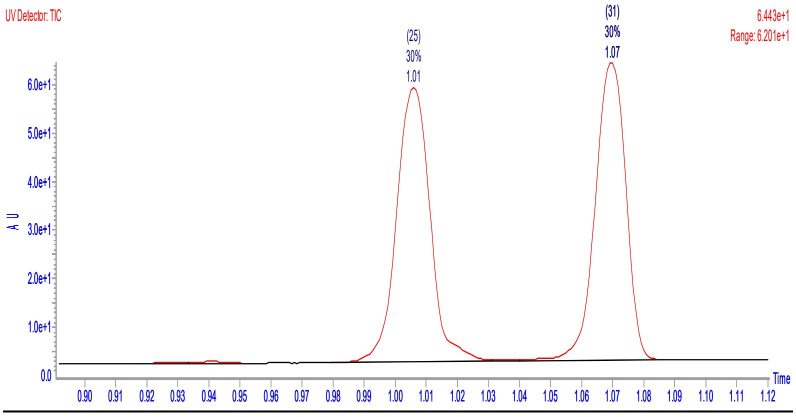

Rt (minutes)	Area (%)
1.01	30
1.07	30

Chiral HPLC: Chiralpak IA (25 × 0.46 cm), 5 μm, *n*-hexane/EtOH = 85/15, 1 ml/min, 220 nm.

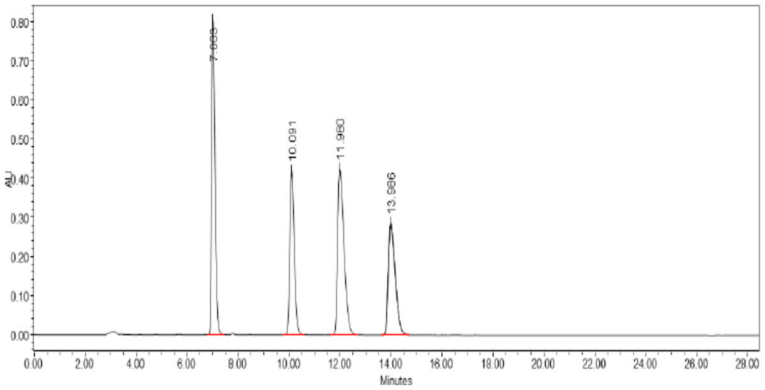

Rt (minutes)	Area (%)
7.0	29.4
11.9	28.8
10.1	20.5
13.9	21.3

Analytical and spectroscopic data for **3af**

(41% yield, *cis*/*trans* = 1/0.6, white solid):

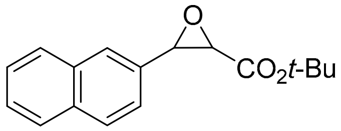

Trans ^{1}H NMR (400 MHz, $CDCl_3$): δ 7.65-7.32 (m, 7H, Ar), 4.2 (d, *J* = 1.3 Hz, 1H), 3.54 (d, *J* = 1.3 Hz, 1H), 1.54 (s, 9H).

Cis ^{1}H NMR (400 MHz, $CDCl_3$): δ 7.65-7.32 (m, 7H, Ar), 4.39 (d, *J* = 4.5 Hz, 1H), 3.8 (d, *J* = 4.8 Hz, 1H), 1.12 (s, 9H).

^{13}C NMR (100 MHz, $CDCl_3$): δ 167.23 (1C, C=O, trans), 165.84 (1C, C=O, cis), 133.57 (1C), 133.2 (1C), 133.04 (1C), 132.73 (1C), 130.72 (1C), 128.57 (1C), 127.96 (1C), 127.85 (1C), 127.8 (1C), 127.73 (1C), 127.64 (1C), 126.56 (1C), 126.47 (1C), 126.28 (1C), 126.17 (1C), 126.08 (1C), 125.96 (1C), 124.24 (1C), 122.59 (1C), 82.77 (1C, *C*H(CH_3)$_3$, *trans*), 82.43 (1C, *C*H(CH_3)$_3$, *cis*), 57.92 (1C, *trans*), 57.51 (1C, *trans*), 57.31 (1C, *cis*), 56.24 (1C, *cis*), 28.03 (1C, CH(*C*H$_3$)$_3$, *trans*), 27.64 1C, (CH(*C*H$_3$)$_3$, *cis*).

MS (ESI, 5600eV): Calcd.: [M+H$^+$]: 271.32; Found: [M+H$^+$]: 271.04.

R$_f$: 0.25 (18/2 Cyclohexane/EtOAc).

Ultra Performance LC analysis:

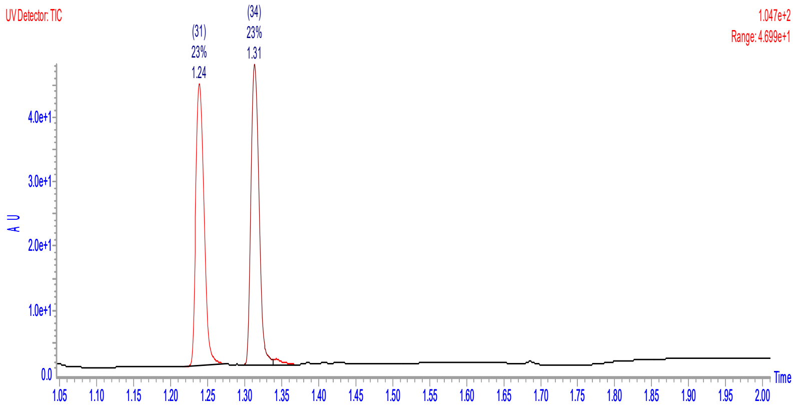

Rt (minutes)	Area (%)
1.24	23
1.31	23

Chiral HPLC: Chiralpak AD-H (25 x 0.46 cm) 5 µm, *n*-hexane/EtOH = 80/20, 1 ml/min, 220 nm.

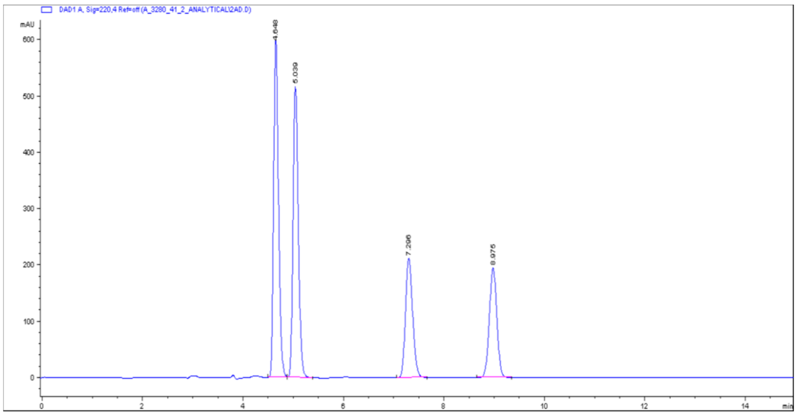

Rt (minutes)	Area (%)
4.6	34
5.0	31.5
7.3	17.5
8.9	17

Analytical and spectroscopic data for **3ce**

(32% yield, *cis*/*trans* = 1/0.9, white solid):

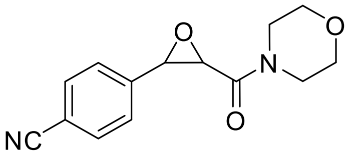

Trans ^{1}H NMR (400 MHz, DMSO-*d6*): 7.87 (d, *J* = 8.3 Hz, 2H), 7.51 (d, *J* = 8.3 Hz, 2H), 4.47 (d, *J* = 4.8 Hz, 1H), 4.24 (d, *J* = 4.8 Hz, 1H), 3.67-2.68 (m, 8H). *Cis* ^{1}H NMR (400 MHz, DMSO-*d6*): 7.84 (d, *J* = 8.5 Hz, 2H), 7.57 (d, *J* = 8.3 Hz, 2H), 4.21 (d, *J* = 1.8 Hz, 1H), 4.17 (d, *J* = 1.8 Hz, 1 H), 3.67-2.68 (m, 8H).

^{13}C NMR (100 MHz, DMSO-*d6*): δ 164.85 (1C, C=O), 163.16 (1C, C=O), 142.02 (1C), 140.62 (1C), 132.89 (2C), 132.5 (2C), 127.71 (2C), 127.65 (2C), 119.1 (1C), 119.08 (1C), 111.7 (1C), 111.42 (1C), 66.53 (1C), 66.5 (1C), 66.39 (1C), 66.37 (1C), 58.32 (1C, *cis*), 56.8 (1C, *trans*), 56.58 (1C, *cis*), 56.31 (1C, *trans*), 45.4 (1C), 44.88 (1C), 42.44 (1C), 41.62 (1C).

MS (ESI, 5600eV): Calcd.: [M+H^{+}]: 259.27; Found:[M+H^{+}]: 259.08.

R$_{f}$: 0.15 (18/2 Cyclohexane/EtOAc).

Ultra Performance LC analysis:

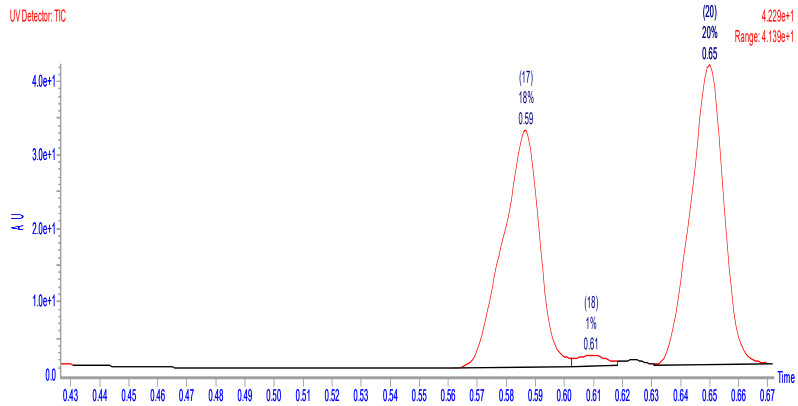

Rt (minutes)	Area (%)
0.59	18
0.65	20

Chiral HPLC: Chiralcel OJ-H (25 x 0.46 cm) 5 µm *n*-hexane/ethanol 60/40, 1 ml/min, 220 nm.

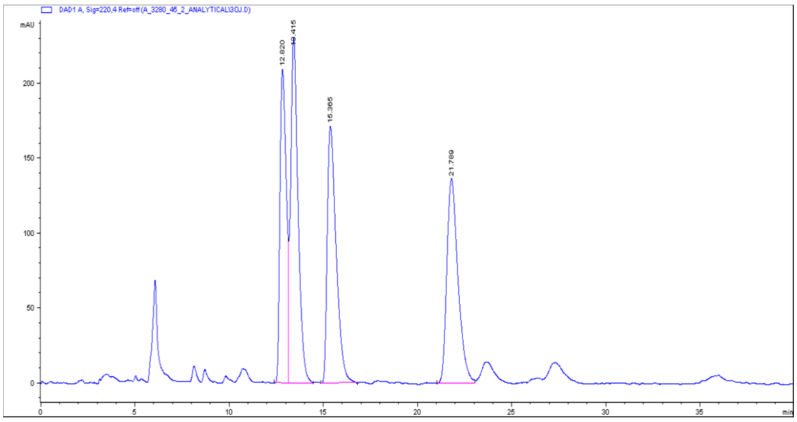

Rt (minutes)	Area (%)
12.8	22
13.4	28
15.3	25
21.7	25

Analytical and spectroscopic data for **3de**

(86% yield, *cis*/*trans* = 1/0.75, white solid):

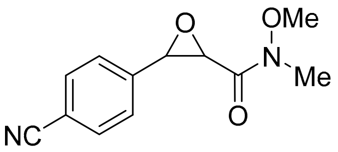

Trans ^{1}H NMR (400 MHz, DMSO-*d6*): δ 7.87 (d, *J* = 8.3 Hz, 2H), 7.6 (d, *J* = 8.3 Hz, 2H), 4.22 (d, *J* = 1.3 Hz, 1H), 4.11 (d, *J* = 1.5 Hz, 1H), 3.69 (s, 3H, Me), 3.18 (s, 3H, Me).

Cis ^{1}H NMR (400 MHz, DMSO-*d6*): δ 7.81 (d, *J* = 8.3 Hz, 2H), 7.55 (d, *J* = 8.3 Hz, 2H), 4.52 (d, *J* = 5 Hz, 1H), 4.35 (bs, 1H), 3.57 (bs, 3H, Me), 2.91 (bs, 3H, Me).

^{13}C NMR (100 MHz, DMSO-*d6*): δ 166.53 (1C, C=O), 165.83 (1C, C=O), 141.91 (1C), 140.34 (1C), 132.97 (2C, *trans*), 132.39 (2C, *cis*), 128.11 (2C, *cis*), 127.59 (2C, *trans*), 119.08 (1C), 119.06 (1C), 111.8 (1C), 111.37 (1C), 62.36 (1C), 62.11 (1C), 57.72 (1C, *cis*), 56.47 (1C, *cis*), 56.27 (1C, *trans*), 56.13 (1C, *trans*), 32.58 (1C), 32.35 (1C).

MS (ESI, 5600eV): Calcd.: [M+H^{+}]: 233.23; Found: [M+H^{+}]: 232.96.

R$_f$: 0.23 (18/2 Cyclohexane/EtOAc).

Ultra Performance LC analysis:

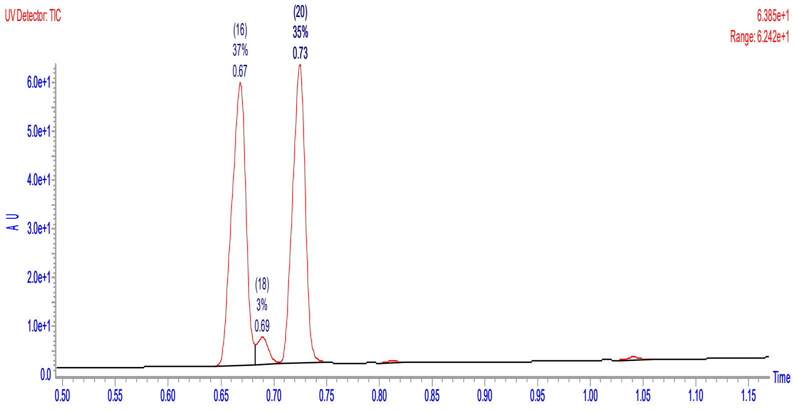

Rt (minutes)	Area (%)
0.67	37
0.73	35

Chiral HPLC: Chiralpak AS-H (25 x 0.46 cm) 5 µm, *n*-hexane/ethanol 70/30, 1 ml/min, 220 nm.

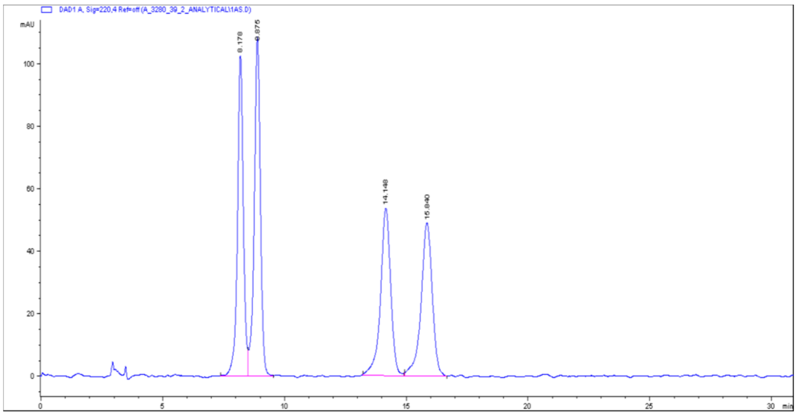

Rt (minutes)	Area (%)
8.1	25.6
8.8	28.1
14.1	23.2
15.8	23.1

^{1}H NMR and ^{13}C NMR Spectra

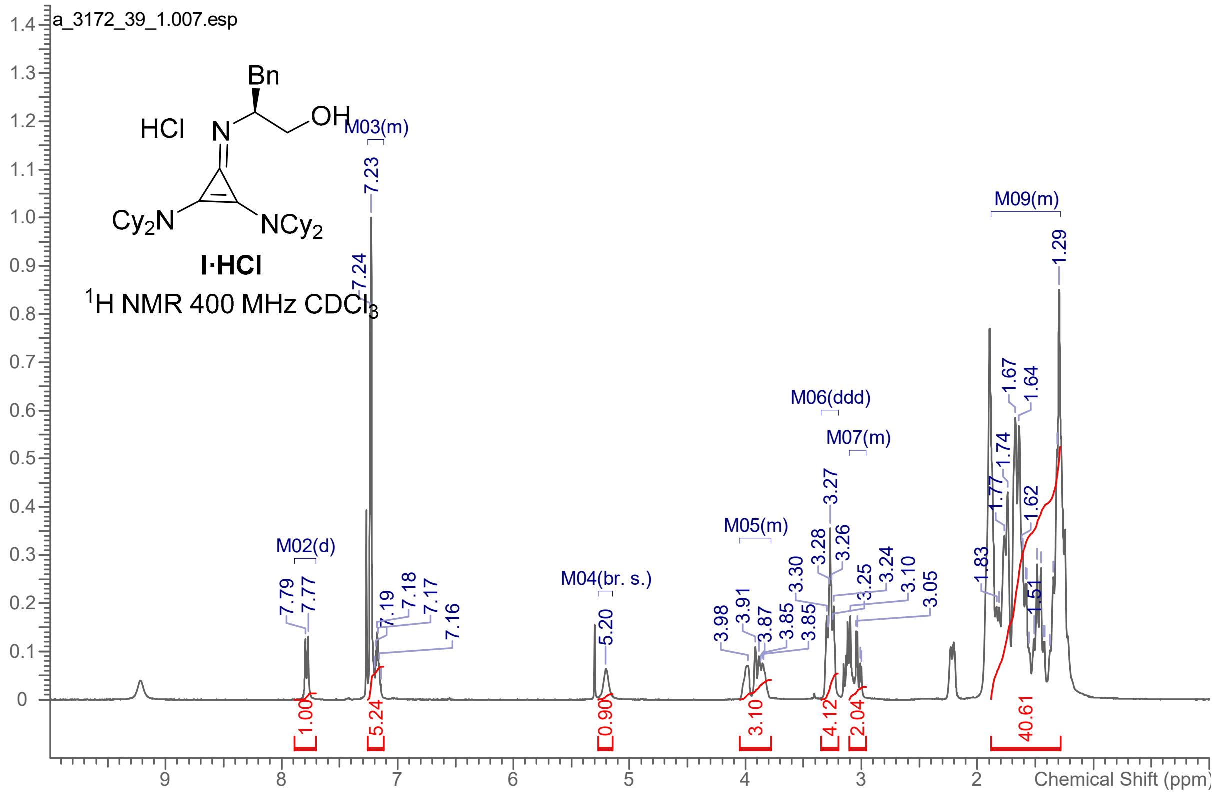

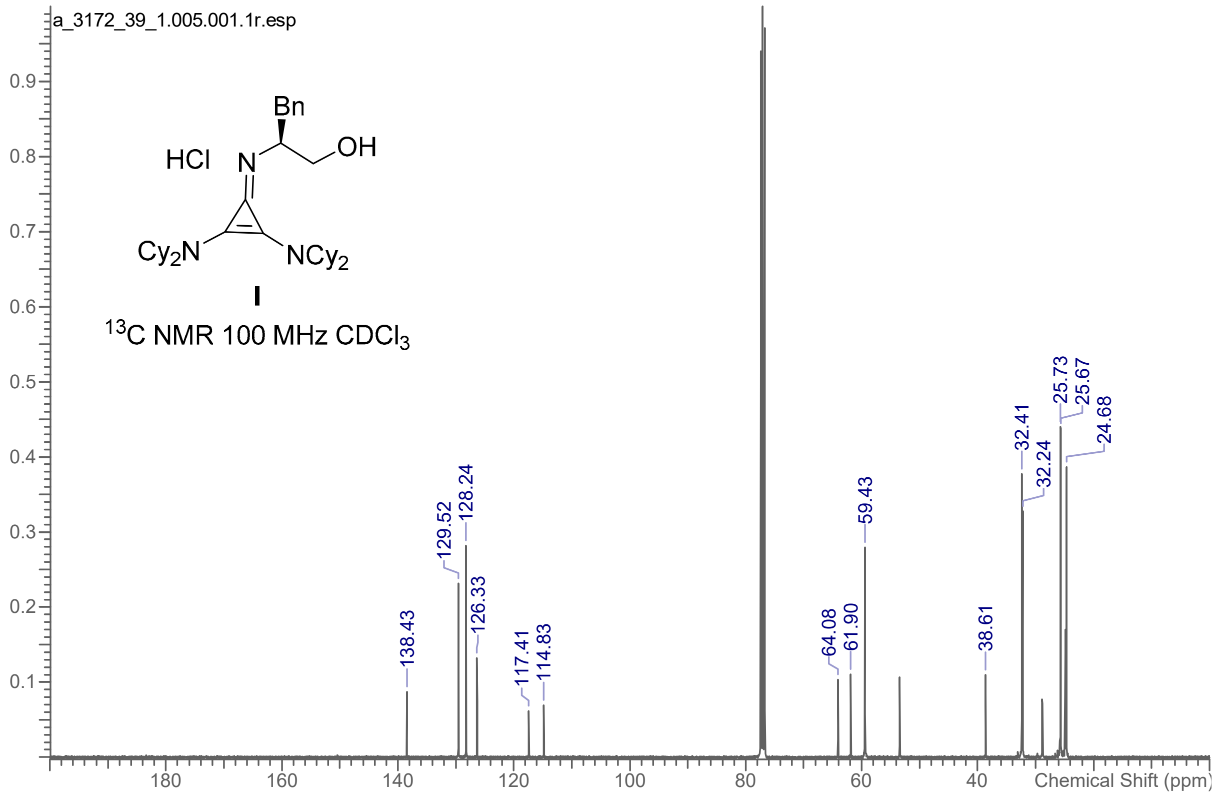

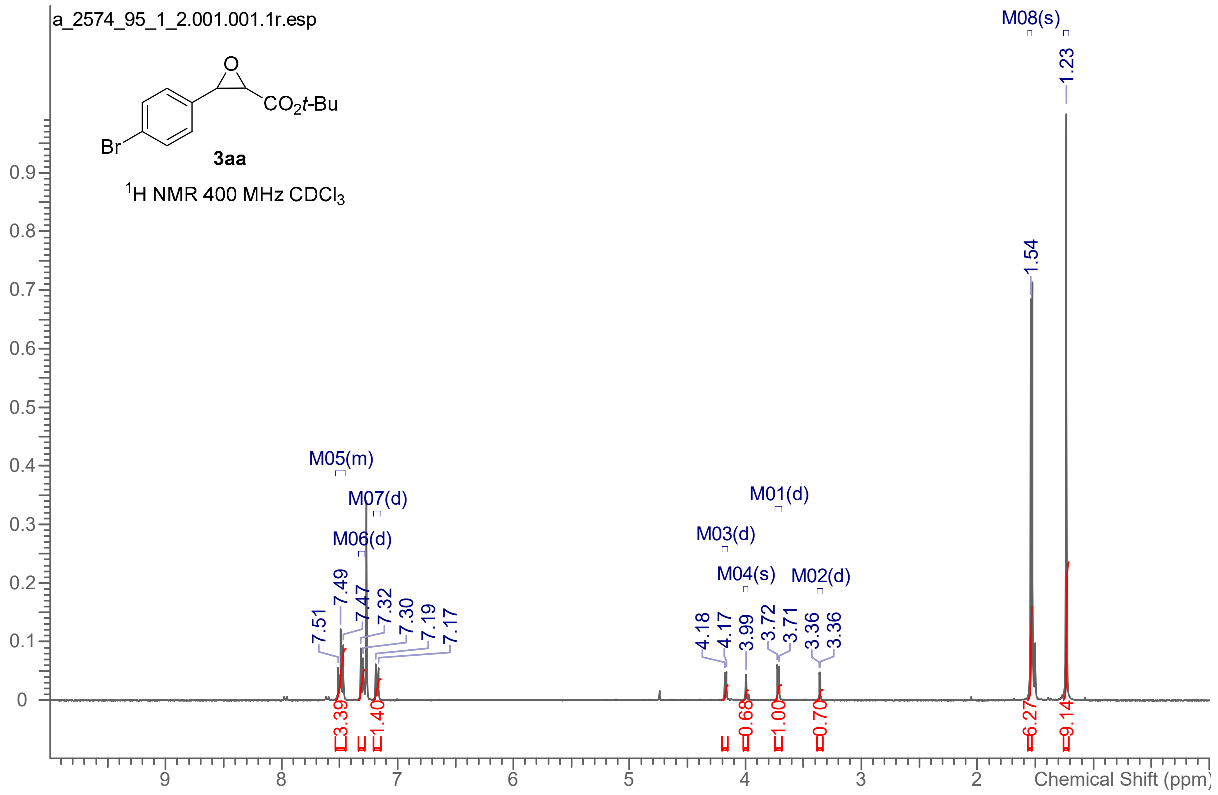
a_2574_95_1_2.001.001.1r.esp
CO2t-Bu
Br
3aa
1H NMR 400 MHz CDCl3
M08(s)
1.23
1.54
M05(m)
M07(d)
M06(d)
M01(d)
M03(d)
M04(s)
M02(d)
7.51
7.49
7.47
7.32
7.30
7.19
7.17
4.18
4.17
3.99
3.72
3.71
3.36
3.36
3.39
1.40
0.68
1.00
0.70
6.27
9.14
Chemical Shift (ppm)

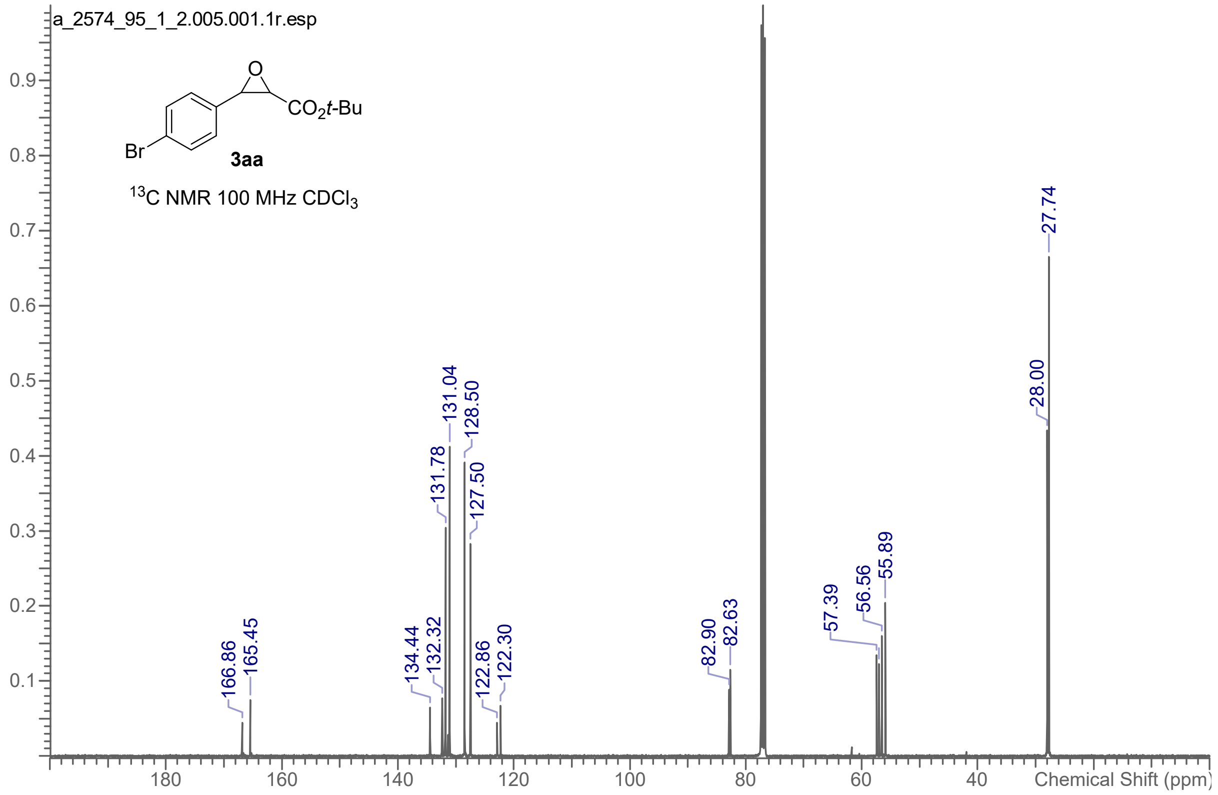
a_2574_95_1_2.005.001.1r.esp
CO2t-Bu
Br
3aa
13C NMR 100 MHz CDCl3
27.74
28.00
131.04
128.50
131.78
127.50
166.86
165.45
134.44
132.32
122.86
122.30
82.90
82.63
57.39
56.56
55.89
Chemical Shift (ppm)

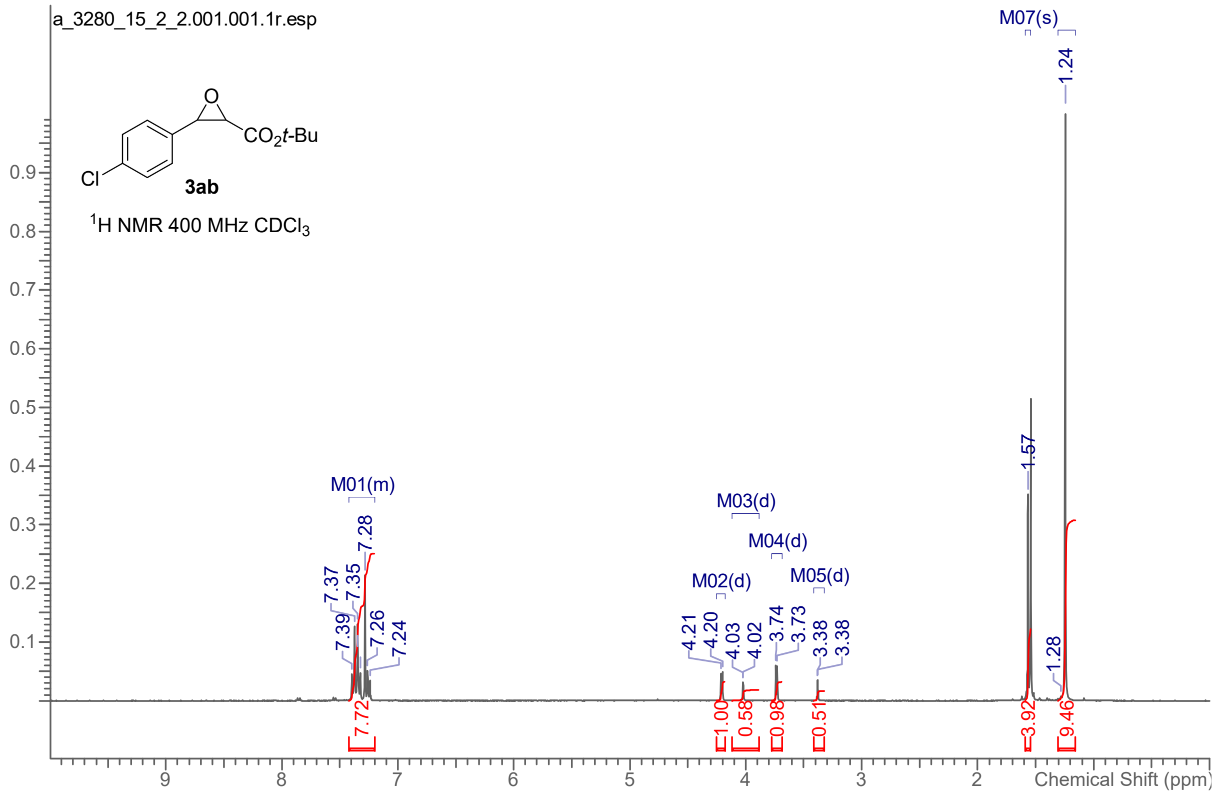
a_3280_15_2_2.001.001.1r.esp
CO2t-Bu
Cl
3ab
1H NMR 400 MHz CDCl3
M01(m)
M02(d)
M03(d)
M04(d)
M05(d)
M07(s)
7.39
7.37
7.35
7.28
7.26
7.24
4.21
4.20
4.03
4.02
3.74
3.73
3.38
3.38
1.57
1.28
1.24
7.72
1.00
0.58
0.98
0.51
3.92
9.46
Chemical Shift (ppm)

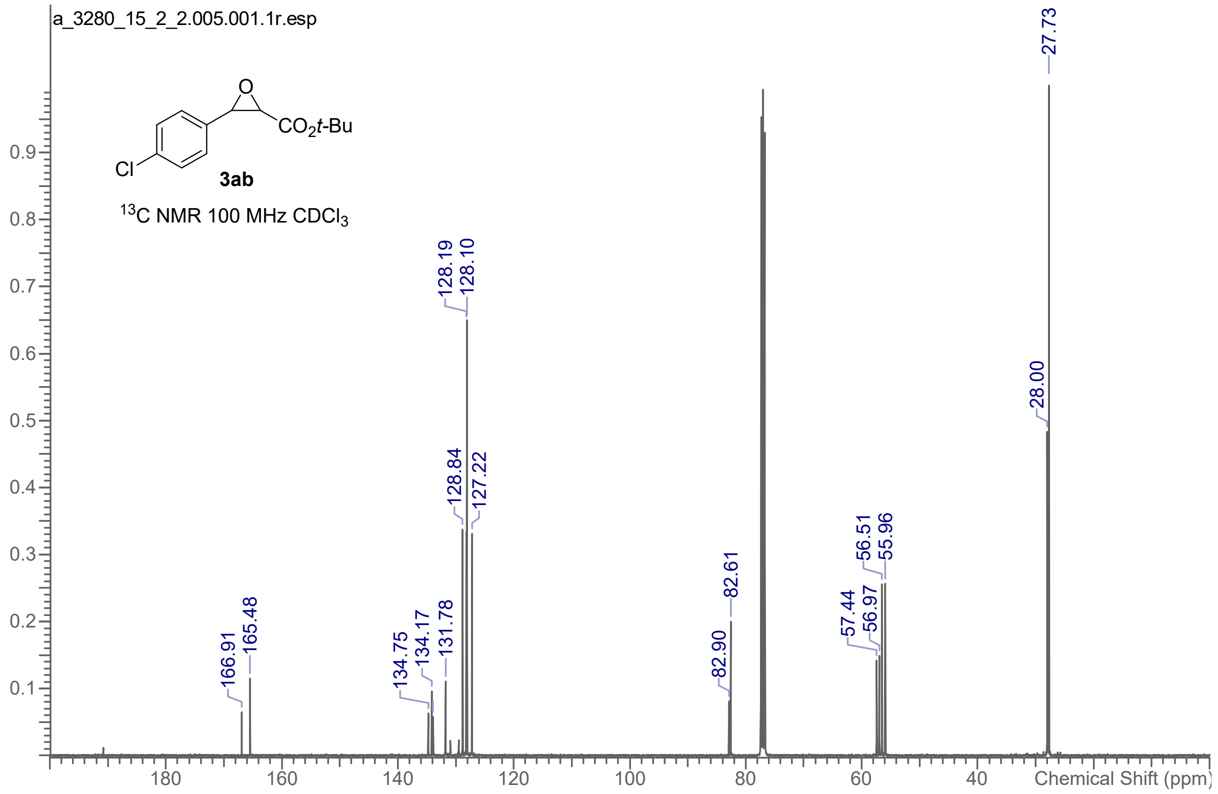
a_3280_15_2_2.005.001.1r.esp
CO2t-Bu
Cl
3ab
13C NMR 100 MHz CDCl3
166.91
165.48
134.75
134.17
131.78
128.84
128.19
128.10
127.22
82.90
82.61
57.44
56.97
56.51
55.96
28.00
27.73
Chemical Shift (ppm)

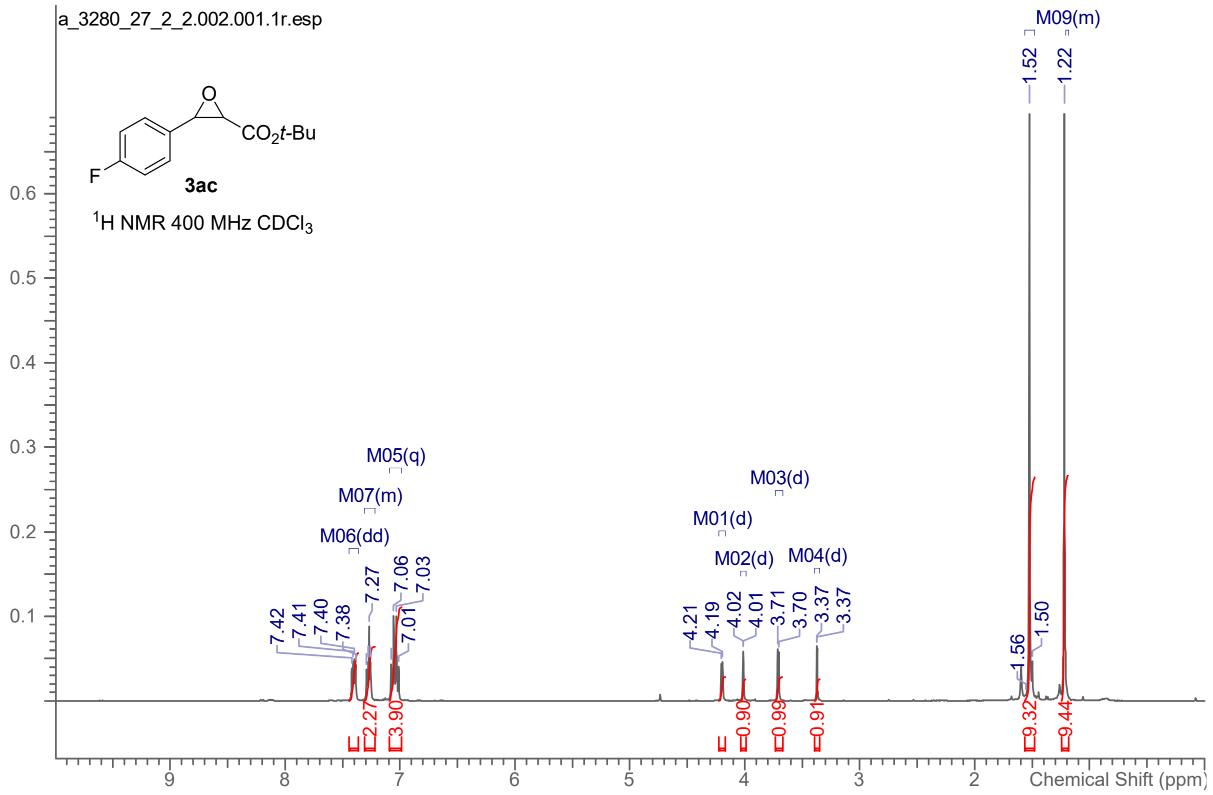
a_3280_27_2_2.002.001.1r.esp
F
O
CO_2t-Bu
3ac
^{1}H NMR 400 MHz $CDCl_3$
M09(m)
1.52
1.22
M05(q)
M07(m)
M06(dd)
7.42
7.41
7.40
7.38
7.27
7.06
7.03
7.01
M03(d)
M01(d)
M02(d)
M04(d)
4.21
4.19
4.02
4.01
3.71
3.70
3.37
3.37
1.56
1.50
2.27
3.90
0.90
0.99
0.91
9.32
9.44
Chemical Shift (ppm)

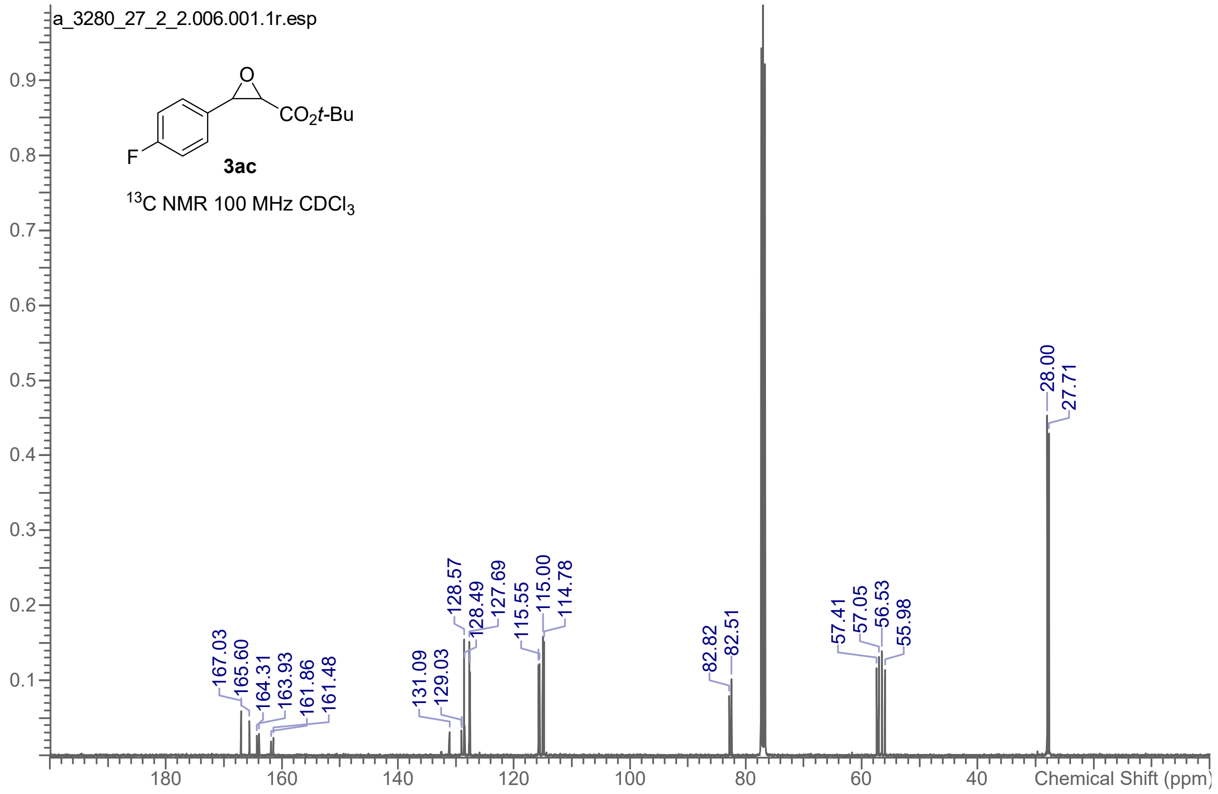
a_3280_27_2_2.006.001.1r.esp
F
O
CO_2t-Bu
3ac
^{13}C NMR 100 MHz $CDCl_3$
167.03
165.60
164.31
163.93
161.86
161.48
131.09
129.03
128.57
128.49
127.69
115.55
115.00
114.78
82.82
82.51
57.41
57.05
56.53
55.98
28.00
27.71
Chemical Shift (ppm)

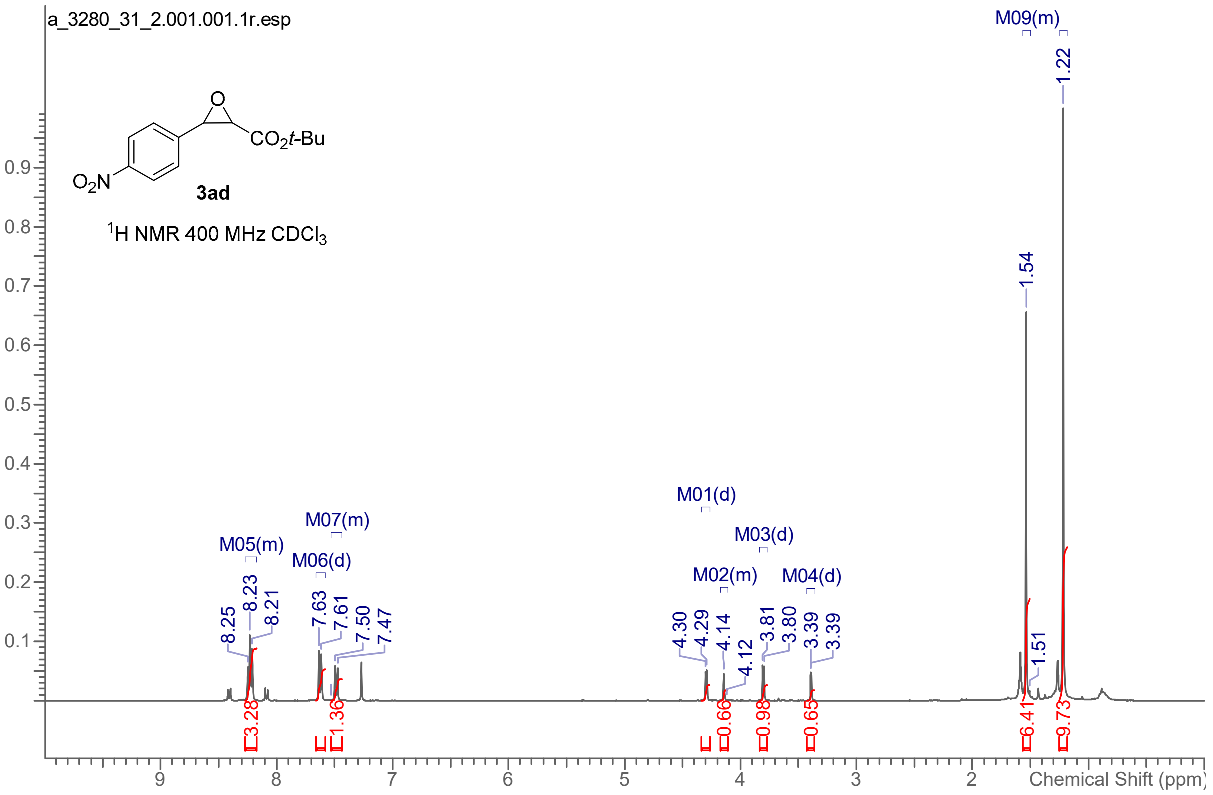
a_3280_31_2.001.001.1r.esp
CO2t-Bu
O2N
3ad
1H NMR 400 MHz CDCl3
M05(m)
M07(m)
M06(d)
M01(d)
M03(d)
M02(m)
M04(d)
M09(m)
8.25
8.23
8.21
7.63
7.61
7.50
7.47
4.30
4.29
4.14
4.12
3.81
3.80
3.39
3.39
1.54
1.51
1.22
3.28
1.36
0.66
0.98
0.65
6.41
9.73
Chemical Shift (ppm)

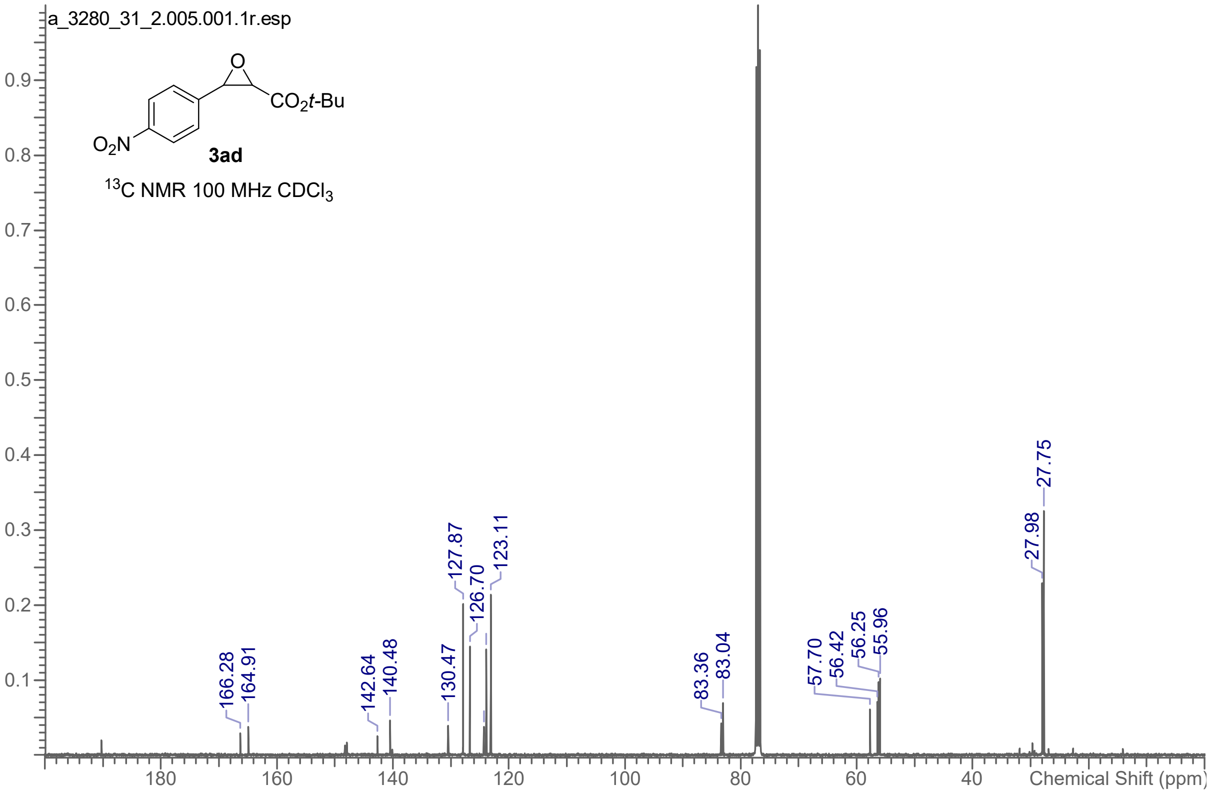
a_3280_31_2.005.001.1r.esp
CO2t-Bu
O2N
3ad
13C NMR 100 MHz CDCl3
166.28
164.91
142.64
140.48
130.47
127.87
126.70
123.11
83.36
83.04
57.70
56.42
56.25
55.96
27.98
27.75
Chemical Shift (ppm)

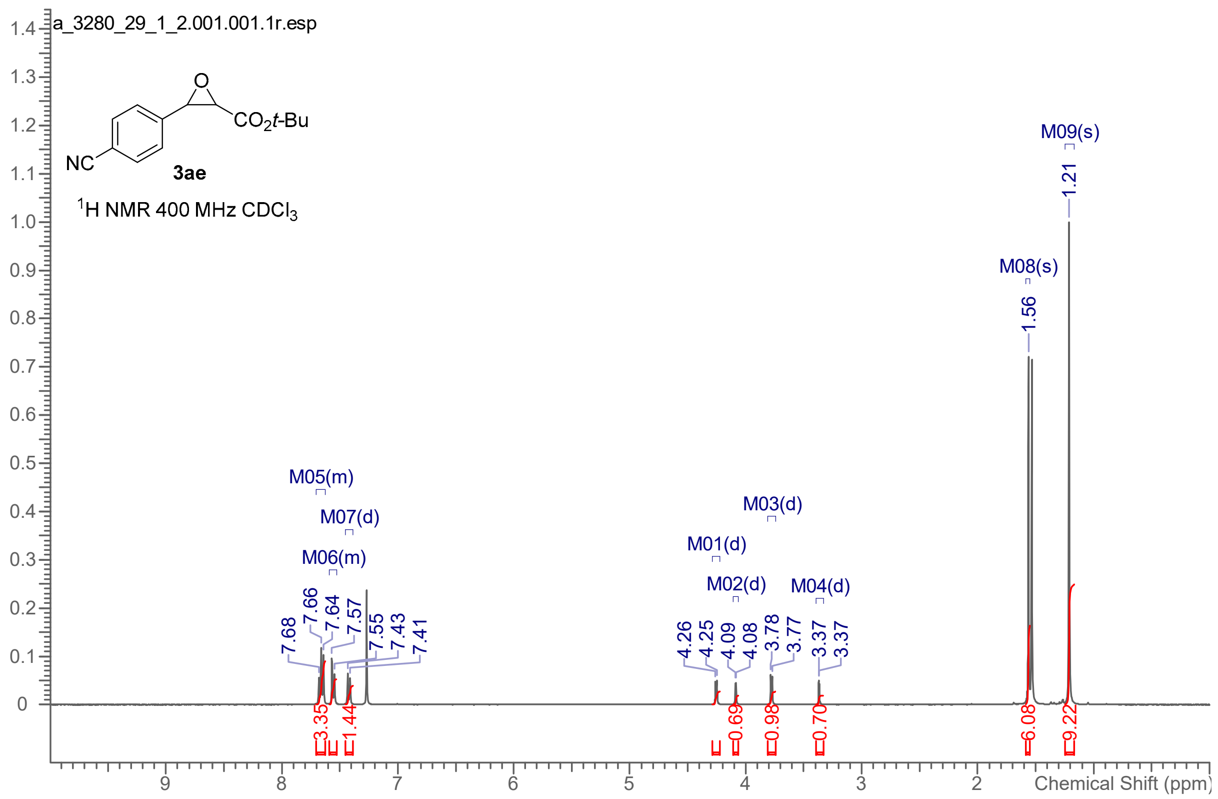
a_3280_29_1_2.001.001.1r.esp
3ae
1H NMR 400 MHz CDCl3
NC
CO2t-Bu
O
M09(s)
M08(s)
M05(m)
M07(d)
M06(m)
M01(d)
M02(d)
M03(d)
M04(d)
7.68
7.66
7.64
7.57
7.55
7.43
7.41
4.26
4.25
4.09
4.08
3.78
3.77
3.37
3.37
1.56
1.21
3.35
1.44
0.69
0.98
0.70
6.08
9.22
Chemical Shift (ppm)

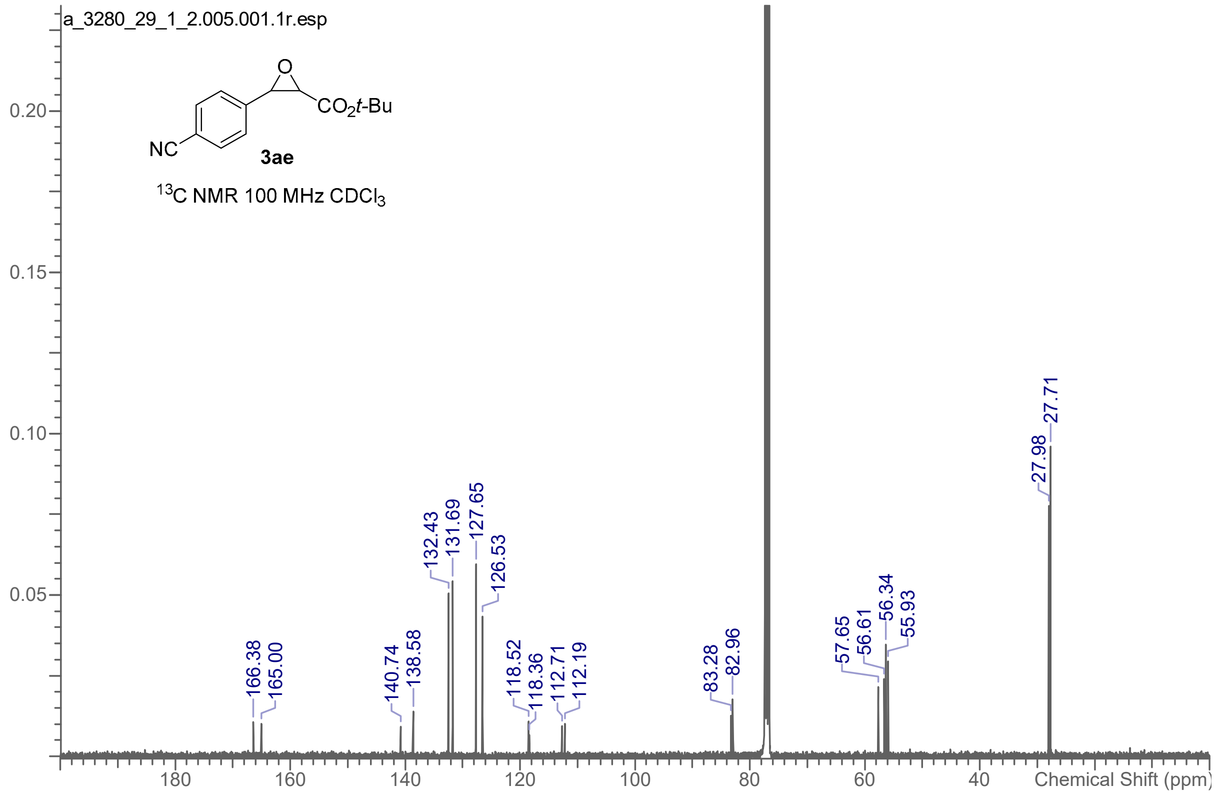
a_3280_29_1_2.005.001.1r.esp
3ae
13C NMR 100 MHz CDCl3
NC
CO2t-Bu
O
166.38
165.00
140.74
138.58
132.43
131.69
127.65
126.53
118.52
118.36
112.71
112.19
83.28
82.96
57.65
56.61
56.34
55.93
27.98
27.71
Chemical Shift (ppm)

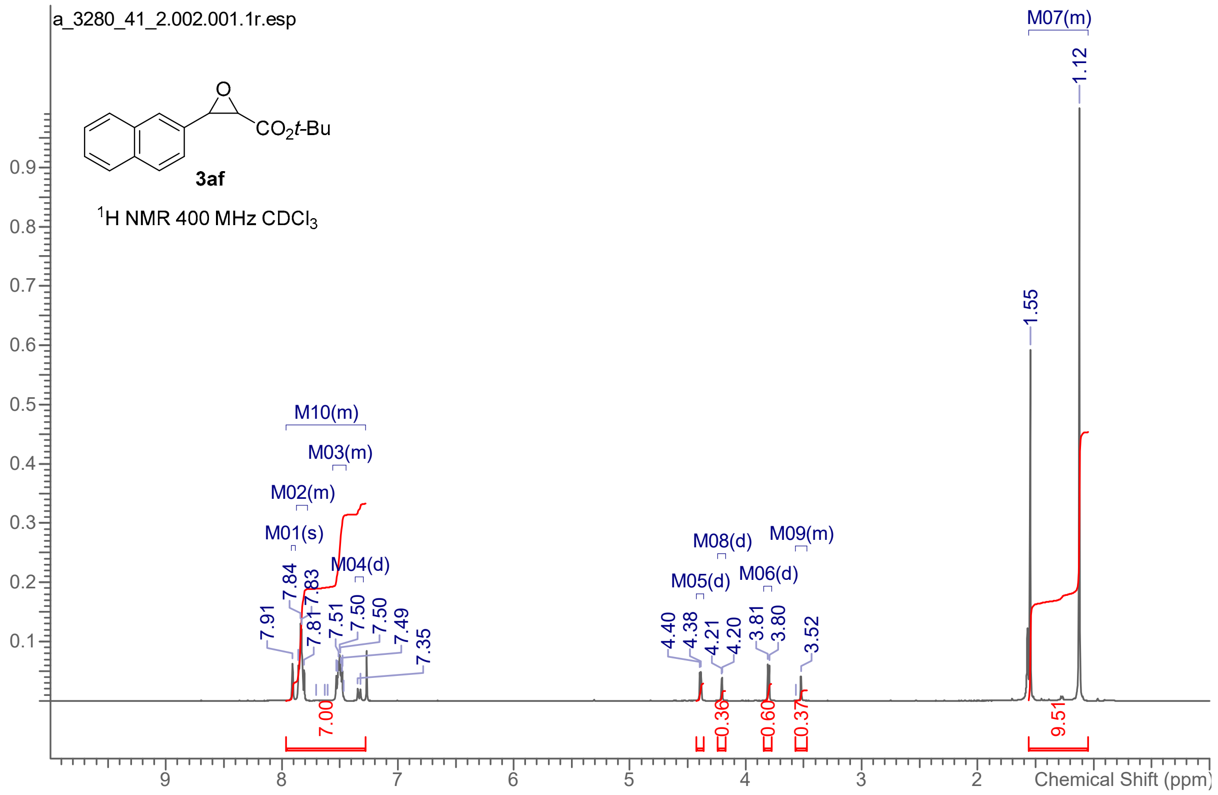
a_3280_41_2.002.001.1r.esp
CO2t-Bu
3af
1H NMR 400 MHz CDCl3
M01(s)
M02(m)
M03(m)
M04(d)
M05(d)
M06(d)
M07(m)
M08(d)
M09(m)
M10(m)
7.91
7.84
7.83
7.81
7.51
7.50
7.50
7.49
7.35
4.40
4.38
4.21
4.20
3.81
3.80
3.52
1.55
1.12
7.00
0.36
0.60
0.37
9.51
Chemical Shift (ppm)

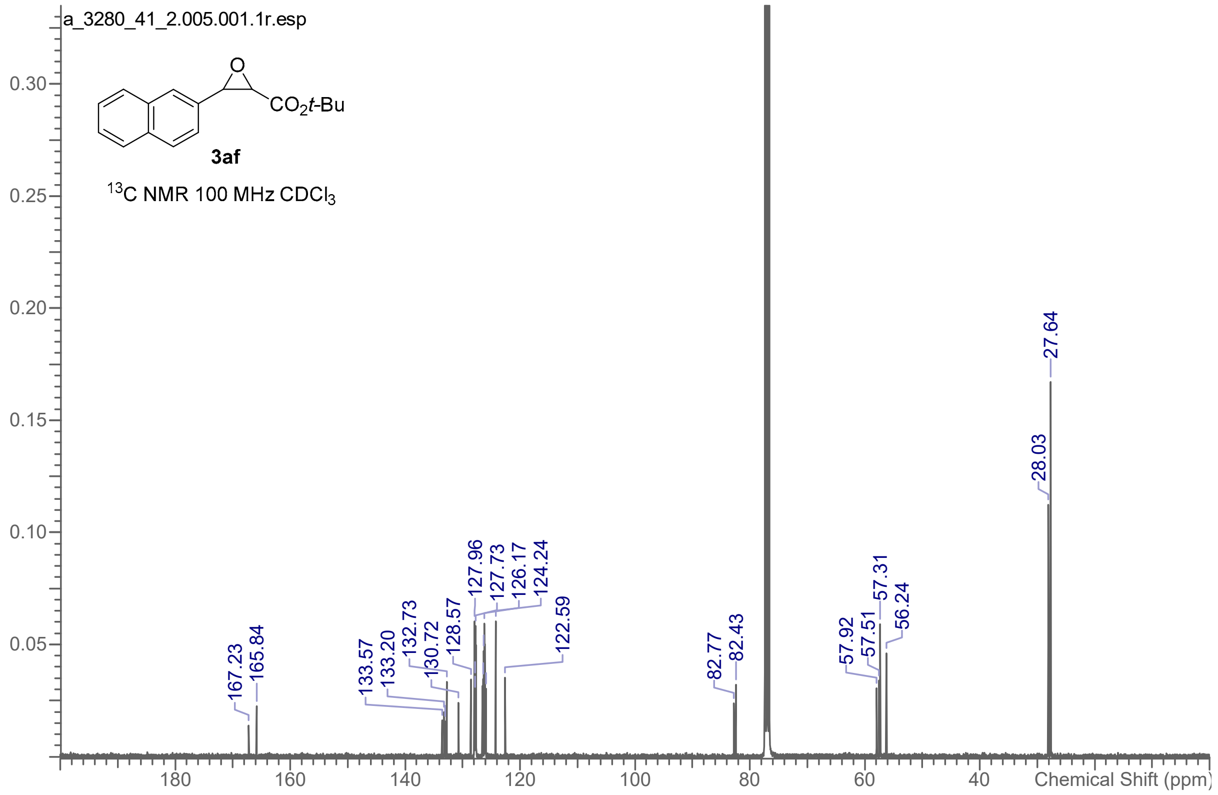
a_3280_41_2.005.001.1r.esp
CO2t-Bu
3af
13C NMR 100 MHz CDCl3
167.23
165.84
133.57
133.20
132.73
130.72
128.57
127.96
127.73
126.17
124.24
122.59
82.77
82.43
57.92
57.51
57.31
56.24
28.03
27.64
Chemical Shift (ppm)

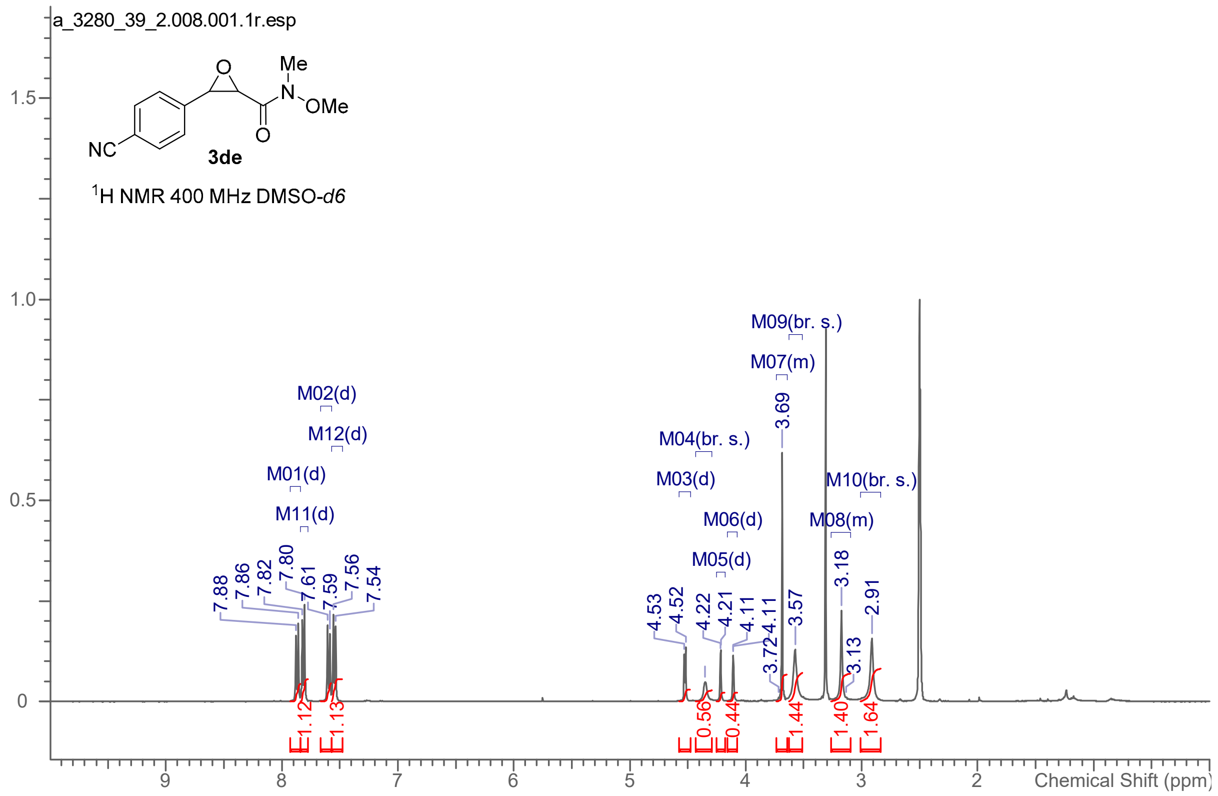
a_3280_39_2.008.001.1r.esp
Me
N
OMe
O
O
NC
3de
^{1}H NMR 400 MHz DMSO-*d6*
M09(br. s.)
M07(m)
M02(d)
M12(d)
M04(br. s.)
M01(d)
M03(d)
M10(br. s.)
M11(d)
M06(d)
M08(m)
M05(d)
7.88
7.86
7.82
7.80
7.61
7.59
7.56
7.54
4.53
4.52
4.22
4.21
4.11
4.11
3.72
3.69
3.57
3.18
3.13
2.91
1.12
1.13
0.56
0.44
1.44
1.40
1.64
Chemical Shift (ppm)

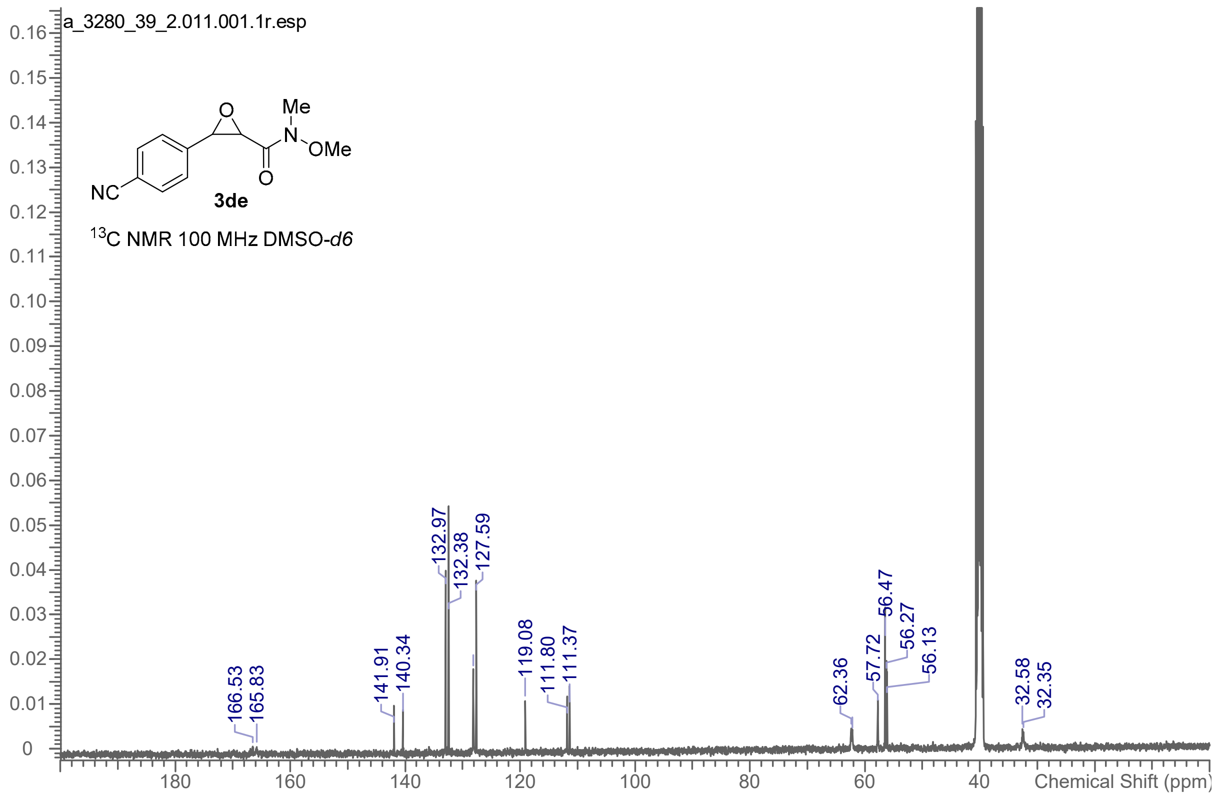
a_3280_39_2.011.001.1r.esp
Me
N
OMe
O
O
NC
3de
^{13}C NMR 100 MHz DMSO-*d6*
166.53
165.83
141.91
140.34
132.97
132.38
127.59
119.08
111.80
111.37
62.36
57.72
56.47
56.27
56.13
32.58
32.35
Chemical Shift (ppm)

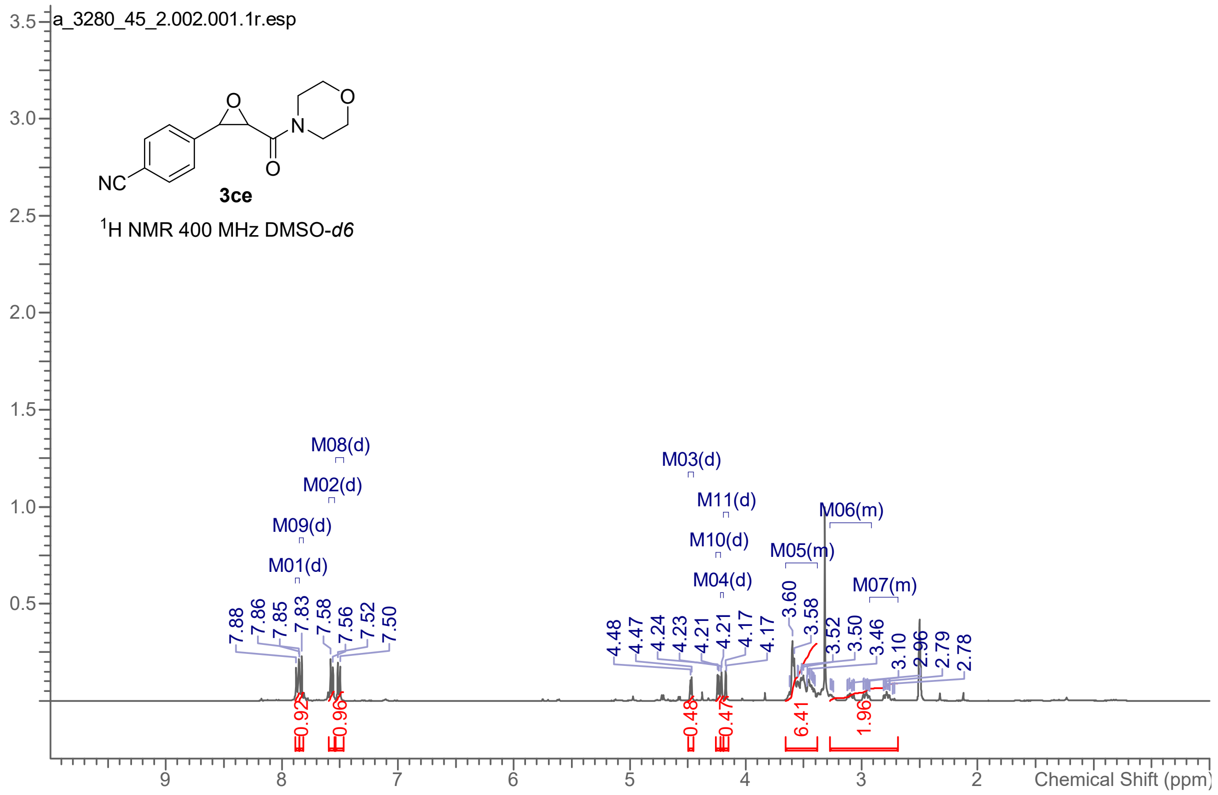
a_3280_45_2.002.001.1r.esp
NC
O
N
O
O
3ce
1H NMR 400 MHz DMSO-d6
M08(d)
M02(d)
M09(d)
M01(d)
M03(d)
M11(d)
M10(d)
M04(d)
M06(m)
M05(m)
M07(m)
7.88
7.86
7.85
7.83
7.58
7.56
7.52
7.50
4.48
4.47
4.24
4.23
4.21
4.21
4.17
4.17
3.60
3.58
3.52
3.50
3.46
3.10
2.96
2.79
2.78
0.92
0.96
0.48
0.47
6.41
1.96
Chemical Shift (ppm)

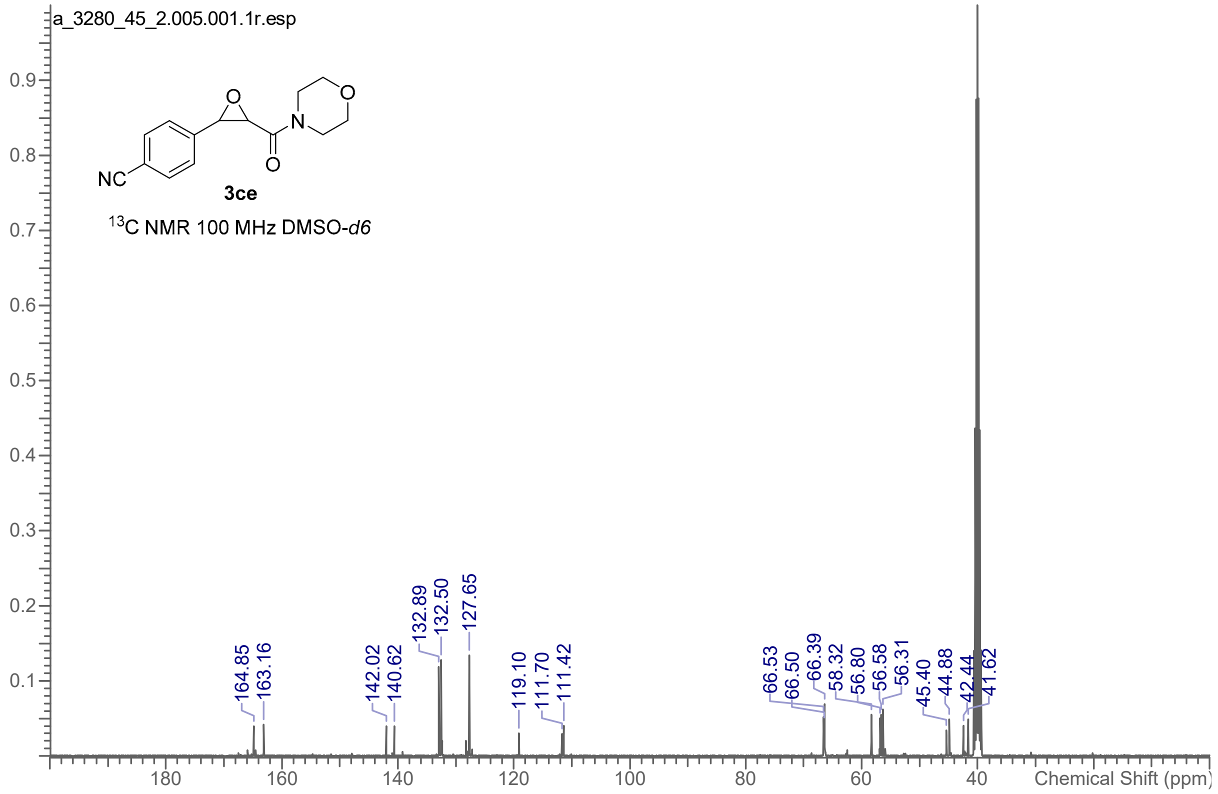
a_3280_45_2.005.001.1r.esp
NC
O
N
O
O
3ce
13C NMR 100 MHz DMSO-d6
164.85
163.16
142.02
140.62
132.89
132.50
127.65
119.10
111.70
111.42
66.53
66.50
66.39
58.32
56.80
56.58
56.31
45.40
44.88
42.44
41.62
Chemical Shift (ppm)

References

[1] Sharifi, A.; Abaee, M. S.; Mirzaei, M.; Salimi, R. Ionic Liquid-Mediated Darzens Condensation: An Environmentally-Friendly Procedure for the Room-Temperature Synthesis of α,β-Epoxy Ketones. *J. Iran. Chem. Soc.* **2008**, *5*, 135-139 (doi.org/10.1007/BF03245826).

[2] Kowalkowska, A.; Jończyk, A. Effect of Phase-Transfer Catalyst on Stereochemistry of tert-Butyl-3-aryl(alkyl)-Substituted Glycidates. *Org. Process Res. Dev*. **2010**, *14*, 728-731. (doi.org/10.1021/op1000379)

[3] Arai, S.; Suzuki, Y.; Tokumaru, K.; Shioiri, T. Diastereoselective Darzens reactions of α-chloroesters, amides and nitriles with aromatic aldehydes under phase-transfer catalyzed conditions. *Tetrahedron Lett.* **2002**, *43*, 833-836.
